# Network meta-analysis of tuina or acupuncture in combination with adjunctive therapy for cervical spondylotic radiculopathy

**DOI:** 10.3389/fneur.2025.1612024

**Published:** 2025-08-08

**Authors:** Shengxiang Zhao, Jie Yang

**Affiliations:** ^1^School of Acupuncture and Tuina, Tianjin University of Traditional Chinese Medicine, Tianjin, China; ^2^Cardiovascular Surgery Department, Tianjin Chest Hospital, Tianjin, China

**Keywords:** cervical spondylotic radiculopathy, tuina, acupuncture, adjunctive therapy, network meta-analysis

## Abstract

**Background:**

Tuina and acupuncture therapy have been widely applied in patients with cervical spondylotic radiculopathy (CSR). This network meta-analysis (NMA) was carried out to compare the effects of tuina or acupuncture in combination with adjunctive therapy on the physical signs, symptoms, and clinical outcomes of patients with CSR.

**Method:**

Relevant studies were searched in PubMed, Web of Science, Embase, Cochrane, China National Knowledge Infrastructure (CNKI), China Science and Technology Journal Database (VIP), Wanfang Data, and China Biology Medicine (CBM), up to June 15, 2023. Randomized controlled trials (RCTs) comparing tuina, acupuncture, or their combination with conventional Western medical adjunctive therapies were selected. Literature quality was assessed using the ROB2 tool, and statistical analyses were conducted using Stata SE15 and R 4.3.1.

**Results:**

90 studies involving 8,612 participants were included. Compared to acupuncture alone, acupuncture + warm needle acupuncture (RR: 17.97; 95% CrI [1.98, 563.78]), acupuncture + cupping (RR: 15.84; 95% CrI [1.48, 538.41]), tuina + auricular acupuncture and conventional therapy (RR: 12.83; 95%CrI [1.31, 170.78]), acupuncture + moxibustion (RR: 8.55; 95% CrI [2.17, 40.28]), and acupuncture + warm needle acupuncture (RR: 8.62; 95% CrI [1.78, 50.25]) significantly improved the clinical response rate, with acupuncture + warm needle acupuncture exhibiting the best effect (SUCRA: 85.9%). Tuina (SUCRA: 75%) ranked highest in improving the cervical function of patients. Electroacupuncture + moxibustion and conventional therapy (SUCRA: 97%) was most effective in relieving pain. None of these therapies effectively improved patient physical signs.

**Conclusion:**

Needling + warm needle acupuncture, warm needle acupuncture + auricular acupuncture, and warm needle acupuncture + conventional therapy may better alleviate symptoms in patients with CSR. However, more well-designed multicenter, large-sample RCTs are needed to further analyze the findings from this study.

**Systematic review registration:**

https://www.crd.york.ac.uk/PROSPERO/view/CRD42023443945, CRD42023443945.

## Introduction

The global prevalence of cervical spondylotic radiculopathy (CSR) is 3.5%, predominantly occurring in individuals aged 40 and older. Due to shifts in socioeconomic factors and alterations in occupational settings, the incidence has risen, increasingly impacting younger demographics (1–3). CSR is a common type of cervical spine disorder; in China, it accounts for 60 to 70% of all cervical spine disorders (4, 5). The primary outcomes included pain indicators (such as Visual Analog Scale [VAS]), physical signs (such as Spurling test), cervical spine function (such as Neck Disability Index [NDI]), and clinical response rate. Secondary outcomes included quality of life (SF-36), analgesic medication usage, and electrophysiological measures (nerve conduction velocity). Although surgery may be required in some cases, CSR is often amenable to non-invasive treatment due to its mechanical and inflammatory features. In addition, the routine clinical integration of adjunctive techniques (such as warm needling and cupping) with core acupuncture and tuina therapies underscores the need for a network meta-analysis (NMA) to evaluate their comparative efficacy. Standard conventional treatments for CSR include physical therapy, pharmacotherapy, psychotherapy, and surgical interventions. Treatment approaches can be employed selectively or in combination, tailored to individual patient conditions and the location of vertebral lesions (6). Conventional pharmacological pain management often involves side effects such as diarrhea and vomiting, and surgical interventions can lead to functional disorders; therefore, conservative treatment has become the preferred approach (7). Moreover, given the complex etiology of cervical disorders, which are classified into various syndromes primarily characterized by compression of the cervical arteries and spinal cord (8), complications such as hypertension, swallowing disorders, and lower limb paralysis are prevalent (9). Pain as well as functional abnormalities of the cervical spine caused by primary symptoms and complications significantly impact the prognosis of patients and negatively affect their quality of life. Therefore, exploring effective conservative treatment measures to alleviate symptoms is crucial for enhancing the quality of life of patients with CSR.

Several clinical studies have demonstrated that therapies such as tuina and acupuncture hold notable significance in conservative treatment owing to their minimal adverse reactions, low recurrence rates, and ease of application. The individualized treatment attributes of acupuncture and tuina facilitate their integration with other Traditional Chinese Medicine (TCM) and Western medical methods, thereby enhancing treatment efficacy (10). Through its distinctive techniques and manual manipulation, tuina aids in alleviating neck muscle tension while enhancing blood circulation and promoting relaxation of the nervous system (11). Acupuncture, by regulating the physiological state of the body’s meridians and nervous system, helps improve the circulation of qi and blood, relaxes muscles and tendons, and relieves symptoms caused by nerve root compression in CSR patients (12). Both treatments exert a positive influence on symptoms related to CSR. These benefits have contributed to the extensive utilization of acupuncture and tuina in CSR treatment, offering patients more comprehensive therapeutic effects. In current clinical practice, a combination of acupuncture and tuina is frequently utilized in conjunction with the 3-step analgesic ladder management approach.

To date, multiple systematic reviews and meta-analyses have reported similar results; however, there remains a lack of standardized clinical recommendations for acupuncture, tuina, and their adjunctive therapies. NMA allows for the comparison of the effects of multiple interventions simultaneously, despite the lack of direct comparisons between these therapies. Furthermore, NMA can rank interventions based on various outcomes, assisting healthcare professionals and clinicians in making evidence-based decisions. Thus, in this study, we selected various TCM therapies with the aim of comparing and ranking their effects on the symptoms and prognosis of CSR patients, while also offering evidence-based recommendations for TCM therapies in CSR patients.

## Methods

### Study design and registration

This NMA was conducted as per the PRISMA statement (13). The study was registered in PROSPERO, with an ID of CRD42023443945.

### Search strategy

Two researchers independently conducted extensive searches on PubMed, Embase, Cochrane, Web of Science, China National Knowledge Infrastructure (CNKI), China Science and Technology Journal Database (VIP), Wanfang Data, and China Biology Medicine disc (CBM), with no restrictions on document type, date/time, or publication status. The search strategy was designed by combining Medical Subject Headings (MeSH) and free text keywords, including all known spellings. Taking PubMed as an example, the search terms were: (Massage[Mesh] OR Acupuncture[Mesh] OR Acupuncture[Title/Abstract] OR Tuina[Title/Abstract]) AND (cervical spondylosis radiculopathy[Title/Abstract] OR ((Spondylosis[Mesh] OR Spondylosis[Title/Abstract]) OR (Lumbarsacral Spondylosis[Title/Abstract]) AND (Radiculopathy[Mesh]) OR Radiculopathies[Title/Abstract])). A detailed search strategy is provided in Appendix 1. Additionally, references to relevant articles were searched for possible supplements. Meanwhile, to prevent literature omission, the databases were re-searched after data extraction.

### Inclusion and exclusion criteria

Inclusion and exclusion criteria were established strictly according to the Population, Intervention, Comparison, Outcomes and Study (PICOS) design. The inclusion criteria are as follows: (1) Patients diagnosed with CSR of any age. (2) Patients in the intervention group received at least one of the following therapies: tuina, acupuncture, or a combination of these interventions. (3) Patients in the control group received a placebo or conventional treatment. If both intervention and control group patients received general adjunctive treatment, it is imperative that the adjunctive treatment be consistent across both groups. (4) Studies that reported at least one of the following outcomes: pain indicators (VAS or Numerical Rating Scale); physical signs (sensory testing by dermatomes, muscle strength grading by manual muscle testing, or provocative tests such as Spurling test); cervical spine function (NDI or Japanese Orthopedic Association scale); or clinical outcomes, defined as overall response rate calculated by (cured + markedly improved + improved) / total cases ×100%. (5) The study adopted a randomized controlled design (i.e., randomized controlled trial, RCT). (6) Studies were limited to those published in English and Chinese.

Considering the applicability to current clinical practice and recommendations, we have selected studies from the past decade to enhance their relevance and significance in research. The exclusion criteria are as follows: (1) Patients with diseases other than CSR. (2) Studies that were not conducted on humans. (3) Case reports, meta-analyses, reviews, guidelines, conference papers, abstracts, and letters. (4) Articles in languages other than English or Chinese. (5) Studies with incompatible interventions.

### Study screening

Based on the pre-defined eligibility criteria, two researchers independently conducted the study screening. Initially, all potentially relevant studies were imported into EndNote X9 to remove duplicates. Subsequently, titles and abstracts were screened to exclude studies that did not meet the defined criteria. Finally, full texts of preliminarily relevant studies were further assessed to confirm the studies to be included.

### Data extraction

Two independent reviewers systematically extracted the following data from eligible studies: (i) study identification: title, first author, and year of publication; (ii) study characteristics: study type (single-center, multicenter, or database-derived), sample size (including proportion of males), participant age (mean ± standard deviation), and disease course; (iii) intervention details: type of intervention (acupuncture or tuina modality), intervention design (treatment group protocol, control group protocol, and adjunctive therapies), treatment period (sessions or duration), and follow-up time; (iv) outcome measures: primary outcomes (pain indicators, physical signs, cervical function, and clinical response rate) and secondary outcomes (quality of life scales, analgesic usage, and electrophysiological parameters). Following independent extraction, reviewers cross-verified all entries. Disagreements were resolved by consensus or third-party adjudication. The same reviewers subsequently evaluated methodological quality using the Cochrane Risk of Bias tool, version 2.0 (RoB 2.0), to assess the bias risk (14). Each study was rated as “low risk of bias,” “some concerns,” or “high risk of bias” in the following domains: bias arising from the randomization process; bias due to deviations from intended interventions; bias due to missing outcome data; bias in measurement of the outcome; and bias due to selective result reporting, including bias associated with deviations from the registered protocol. If one or more domains were assessed as “high risk of bias,” the study was rated as high risk of bias. Conversely, if all domains were assessed as “low risk of bias,” it was rated as low risk of bias.

### Data analysis and quality assessment

A Bayesian framework-based statistical model was constructed using the JAGS (gemtc package 0.8–2 and rjags package 4–10) in R (v4.1.2) (Rstudio, Boston, MA, United States). Mean differences (MD) and 95% credible intervals (CrI) were calculated for continuous data to determine the size of the effect. For categorical data, the pooled risk ratio (RR) with its 95% CrI was calculated. Given the clinical heterogeneity of the included trials (differing acupuncture durations, needle placement, sample sources, and patient disease course), a random-effects model was adopted for all NMAs. The surface under the cumulative ranking curve (SUCRA) was used to estimate the relative rank of different interventions for each relevant outcome (15). Higher SUCRA values indicated a higher ranking of the intervention (15). Additionally, the Deviance Information Criterion (DIC) was adopted to compare the consistency model with the inconsistency model. A DIC difference of less than 5 points indicated good consistency, and the consistency model would be adopted (16). Comparison-adjusted funnel plots were used to test for publication bias. The network diagrams and comparison-adjusted funnel plots for the NMA were drawn using Stata (v15.0) (StataCorp, College Station, Texas, United States).

## Results

### Search results

**Figure 1:**
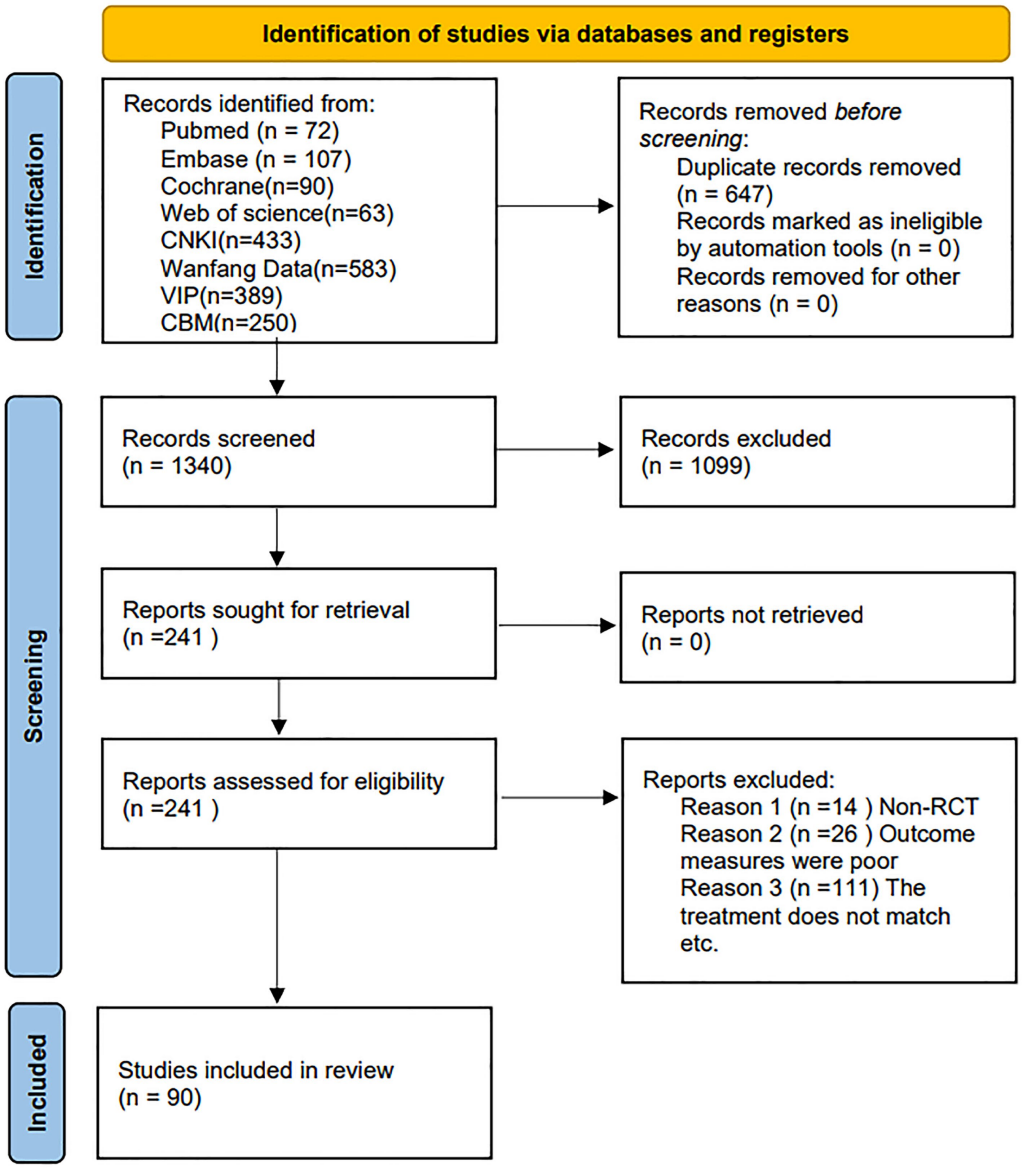
PRISMA flowchart for search and screening of eligible studies in the NMA.

The PRISMA flowchart detailed in Figure 1 illustrates the comprehensive process of study screening. Initially, a total of 1,987 potentially relevant studies were identified from the eight electronic databases mentioned. After removing 647 duplicates, the titles and/or abstracts of the studies were screened based on the pre-defined eligibility criteria, leading to the exclusion of 1,099 studies. The full texts of the remaining 241 studies were then reviewed for further assessment of eligibility, resulting in the exclusion of 14 studies due to non-compliance with RCT standards, 26 studies due to inadequate outcome assessment criteria, and 111 studies due to incompatible treatment methods (details are presented in Figure 1). Ultimately, 90 studies were deemed eligible and included in the NMA.

### Characteristics of included studies

The characteristics and details of each study included in the NMA are detailed in Appendix 2. The references for these articles are provided in Appendix 3. All 90 eligible studies published between 2013 and 2023 were conducted in Asia. In general, these 90 RCTs involved 8,612 CSR patients, with sample sizes ranging from 27 to 300, and participant ages ranging from 18 to 93 years. Interventions in the studies varied, with acupuncture being used in 10 studies, tuina in 5 studies, both acupuncture and tuina in 15 studies, and combinations of acupuncture or tuina with other adjunctive therapies in 60 studies.

### Quality assessment

**Figure 2:**
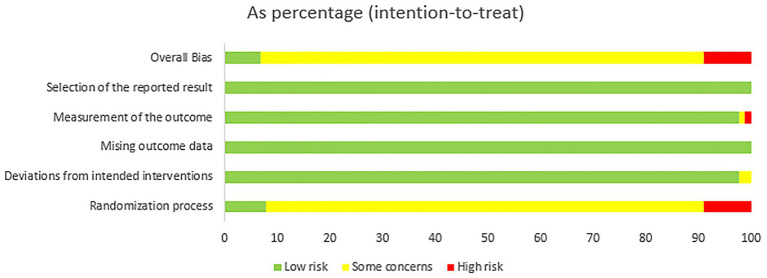
Analysis of the risk of bias in the literature.

**Figure 3:**
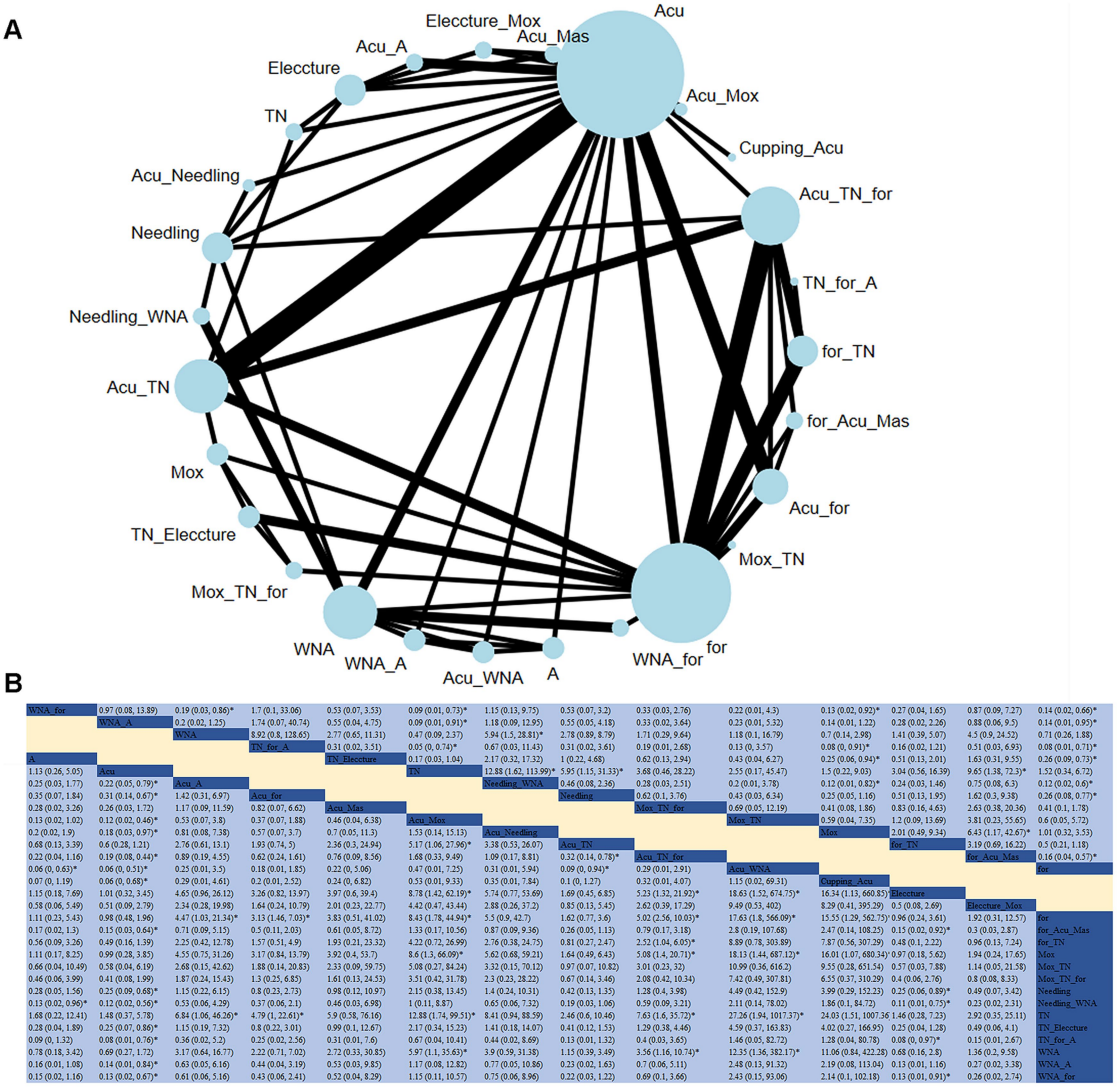
**(A)** Network diagram of clinical response (case). **(B)** Relative impact of different therapeutic methods on clinical response.

The results of the risk of bias assessment are presented in Figures 2, 3. The majority of the RCTs we a low risk of bias in the domains of selection of the reported results (*n* = 90.100%), missing outcome data (*n* = 90.100%), measurement of the outcome (*n* = 88.98%), and deviations from intended interventions (*n* = 88.98%). However, a high proportion of studies raised concerns about bias during the randomization process (*n* = 83.92%). The main areas of high risk were due to issues with randomization and outcome measurement.

### Network meta-analysis

#### Clinical response

A total of 49 studies evaluated the impact of 27 therapeutic methods on the clinical response of CSR (Figure 3A). Compared to acupuncture alone, acupuncture + warm needle acupuncture (RR: 17.97; 95% CrI [1.98, 563.78]), acupuncture + cupping (RR: 15.84; 95% CrI [1.48, 538.41]), tuina + auricular acupuncture and conventional therapy (RR: 12.83; 95% CrI [1.31, 170.78]), acupuncture + moxibustion (RR: 8.55; 95% CrI [2.17, 40.28]), and needling + warm needle acupuncture (RR: 8.62; 95% CrI [1.78, 50.25]) significantly improved the clinical response rate. Compared with acupuncture + tuina, acupuncture + warm needle acupuncture (RR: 10.84; 95% CrI [1.06, 347.69]), and acupuncture + moxibustion (RR: 5.17; 95% CrI [1.06, 27.96]) significantly improved the clinical response rate. Based on the SUCRA, ACU_WNA was considered the most effective intervention for improving clinical response (SUCRA = 85.9%).

#### Pain

**Figure 4:**
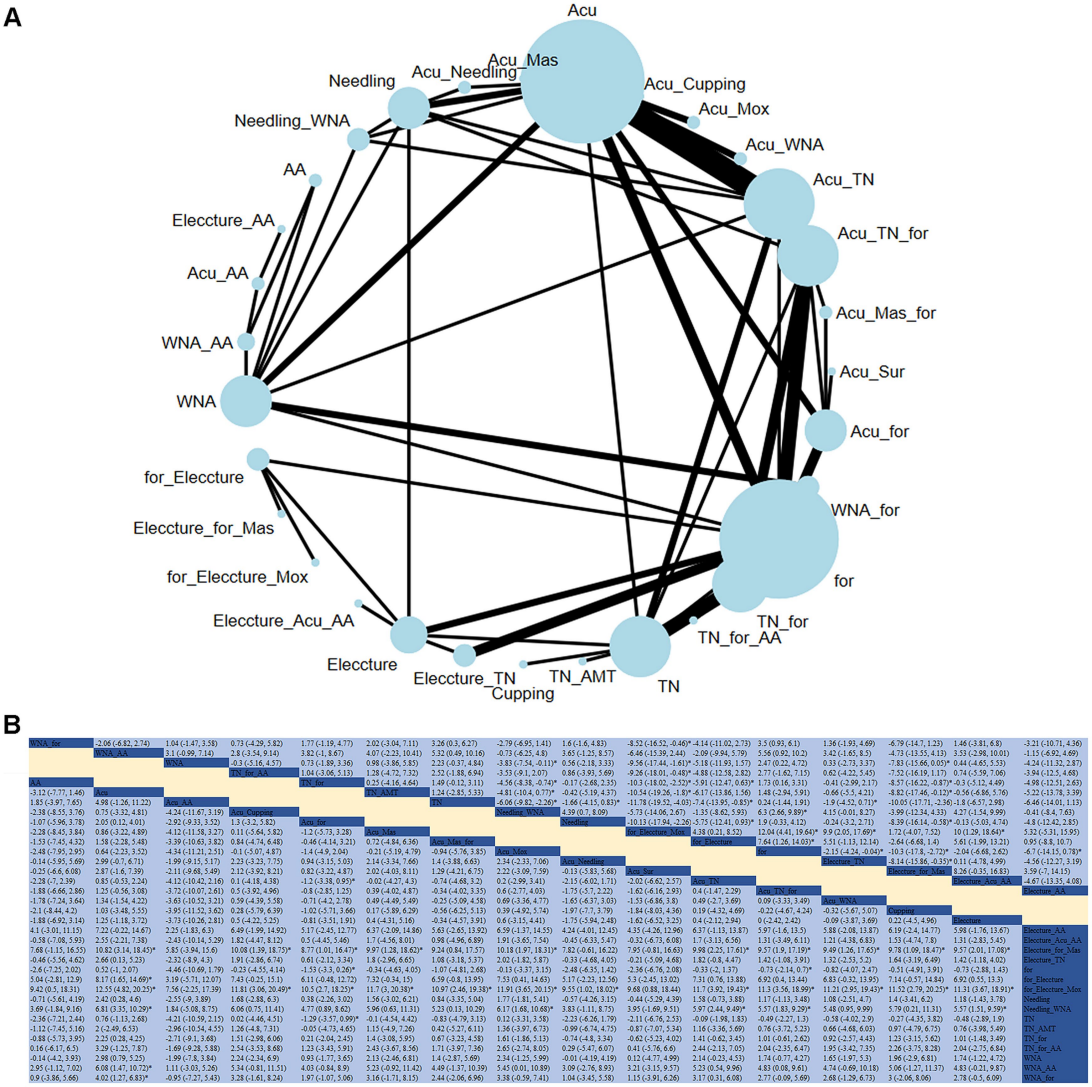
**(A)** Network diagram of pain indicators. **(B)** Relative impact of different treatment methods on pain.

An NMA was conducted on 66 studies to evaluate the effects of acupuncture or tuina and their adjunct therapies on patient pain conditions (Figure 4). Compared to acupuncture alone, significant improvements in pain conditions were observed with the combination of electroacupuncture + moxibustion and conventional therapy (RR: −12.55; 95% CrI [−20.25, −4.82]), electroacupuncture + massage and conventional therapy (RR: −10.82; 95% CrI [−18.45, −3.14]), electroacupuncture + conventional therapy (RR: −8.17; 95% CrI [−14.69, −1.65]), needling + warm needle acupuncture (RR: −6.81; 95% CrI [−10.29, −3.35]), and warm needle acupuncture + auricular acupuncture (RR: −6.08; 95%CrI [−10.72, −1.47]). Based on the SUCRA, for_Eleccture_Mox was considered the most effective intervention for improving clinical response (SUCRA = 97.1%).

#### Cervical spine function

**Figure 5:**
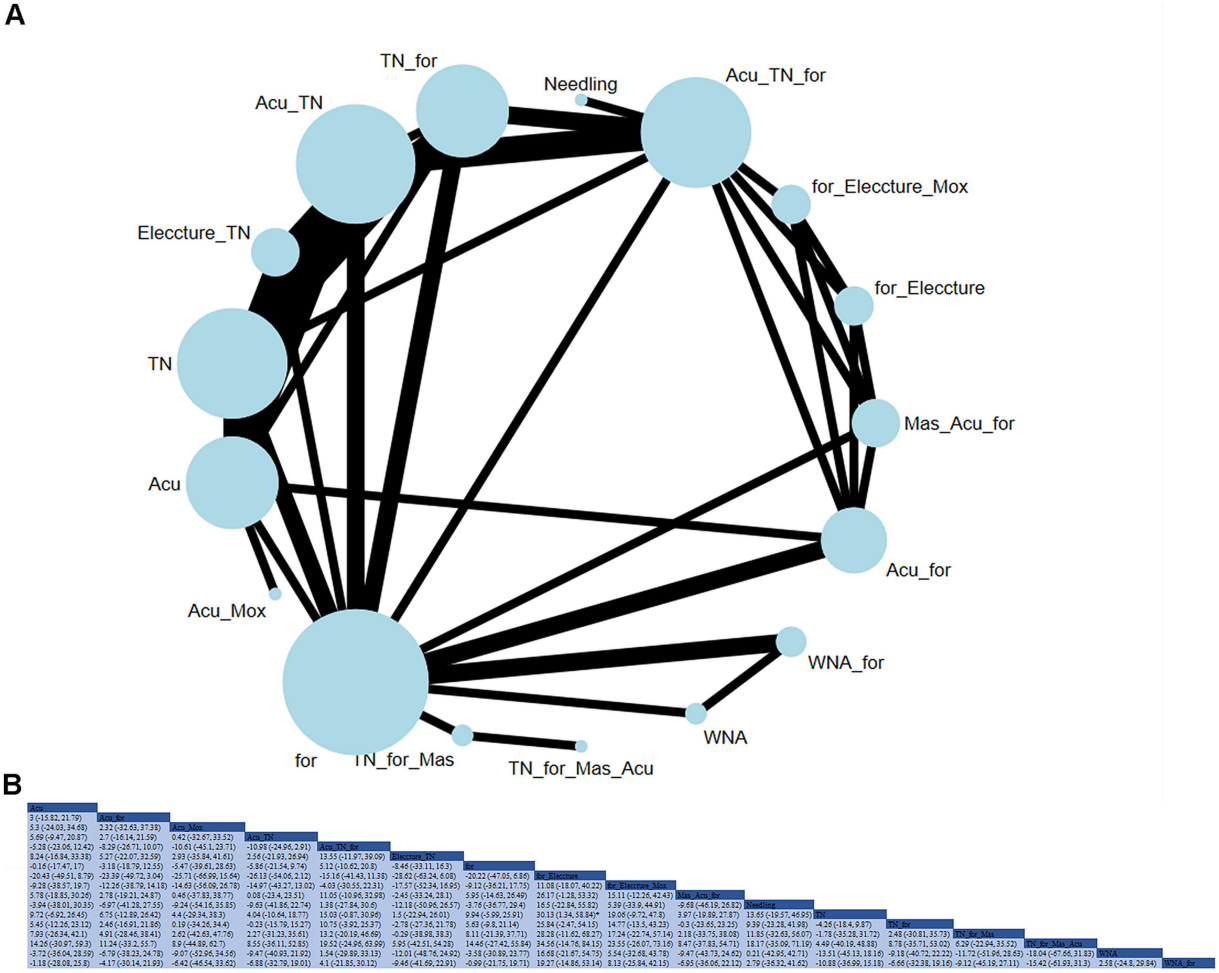
**(A)** Network diagram for cervical spine function (JZ). **(B)** Relative impact of different treatment methods on cervical spine function.

An NMA analysis was carried out on 26 studies to evaluate the effect of acupuncture or tuina and their adjunct therapies on cervical spine function (Figure 5). Compared to tuina alone, electroacupuncture + conventional therapy significantly aided in the recovery of cervical spine function (RR: −30.13; 95% CrI [−58.84, −1.34]). Based on SUCRA, tuina was considered the most effective intervention for improving cervical spine function (SUCRA = 75.1%).

#### Physical signs

**Figure 6:**
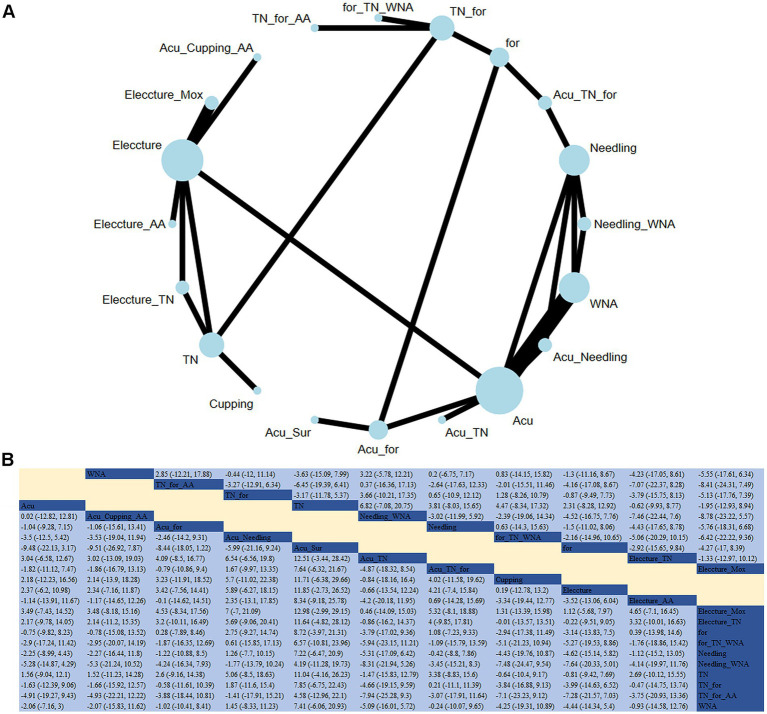
**(A)** Network diagram for symptoms (ZZ). **(B)** Relative impact of different treatment methods on cervical spine function.

An NMA analysis was carried out on 22 studies to evaluate the effect of acupuncture or tuina and their adjunct therapies on physical signs (Figure 6). As per the league table, pairwise comparisons between interventions were not statistically significant. Based on SUCRA, Eleccture_Mox was considered the most effective intervention for improving cervical spine function (SUCRA = 77.1%).

#### Safety analysis

Based on the RCTs included, the common adverse reactions were as follows:

- Acupuncture: localized pain or soreness during needle insertion or retention; minor bleeding or subcutaneous bruising caused by microvascular injury; localized redness or mild allergic reactions, most commonly to disinfectants and, rarely, to the needle material.
- Tuina: post-treatment soreness in the manipulated area, resembling exercise-induced muscle soreness, typically resolving within 1–2 days; localized skin redness or minor abrasions, especially in individuals with sensitive skin.
- Needling fainting: vasovagal reactions (dizziness, nausea, pallor, sweating, and syncope) triggered by anxiety, hunger, fatigue, or strong stimulation. These events are preventable with proper patient positioning (preferably supine), avoidance of fasting, and timely clinical intervention.
- Stuck or bent needles: difficulty in needle removal (stuck needle) or needle deformation, typically due to patient movement or improper technique. These incidents require prompt practitioner intervention.
- Transient symptom exacerbation: a brief worsening of pre-existing symptoms (such as pain or numbness) following tuina or acupuncture, usually resolving within hours to 2 days. Although considered a therapeutic response rather than an adverse event, it must be clearly distinguished from actual injury.
- Transient nerve irritation: temporary aggravation of radicular symptoms (such as numbness or pain) caused by deep needling or inappropriate tuina technique (such as excessive traction or rotation).

While the risk of permanent nerve injury is extremely low when proper technique is employed, it remains a specific concern in procedures such as the treatment of cervical radiculopathy. Details on the safety outcomes and adverse reactions are provided in Appendix 4.

#### Heterogeneity and publication bias assessment

The DIC was employed to compare the consistency and inconsistency models. All closed-loop models demonstrated variations smaller than 5, indicating good consistency with the DIC. The absolute DIC values and their differences were as follows: (i) clinical response: consistency model DIC = 189.59, inconsistency model DIC = 185.35 (ΔDIC = 4.24); (ii) pain: consistency model DIC = 276.82, inconsistency model DIC = 277.45 (ΔDIC = 0.63); (iii) physical signs: consistency model DIC = 93.89, inconsistency model DIC = 93.99 (ΔDIC = 0.10); (iv) cervical spine function: consistency model DIC = 109.90, inconsistency model DIC = 109.92 (ΔDIC = 0.02). All closed-loop models demonstrated ΔDIC values below 5 (range: |0.02| to |4.24|), indicating good overall consistency across the network. In terms of publication bias assessment, comparison-adjusted funnel plots showed no evidence of publication bias (Supplementary Figures S1–S4).

## Discussion

In this study, a comprehensive search was conducted for relevant publications, and the available evidence from 90 NMA RCTs was analyzed. The analysis aimed to compare the effects of acupuncture or tuina combined with other adjunct therapies on the clinical efficacy, pain, physical signs, and cervical spine function in patients with CSR. The studies indicated significant clinical benefits in the treatment of CSR patients with the following methods: acupuncture + warm needle acupuncture greatly enhanced clinical treatment outcomes; electroacupuncture and moxibustion + conventional therapy effectively alleviated pain in CSR patients; additionally, electroacupuncture + moxibustion effectively improved physical signs in CSR patients; tuina therapy, meanwhile, showed the most notable improvement in cervical spine function in CSR patients.

Previous meta-analyses (17–25) indicated that some adjuvant therapies combined with acupuncture or tuina better alleviated adverse conditions in CSR patients, aligning with our findings.

In terms of clinical response, certain studies (26, 27) have indicated that both massage and tuina can effectively enhance the clinical response in CSR patients, possibly attributed to their ability to regulate muscle tension at rest by ameliorating muscle edema and adhesion, thereby restoring neck function (26). Several viewpoints (28–30) suggested that, during tuina therapy, there is a decrease in serum levels of TNF-α, IL-6, and IL-1β in patients, which suppresses inflammatory responses, improves clinical efficacy, and concurrently increases nitric oxide synthesis while reducing endothelin (ET) release, thereby alleviating vertebral artery spasm and mitigating patient symptoms. Prior studies (19, 21, 22, 25) indicated that compared to conventional acupuncture or simple traction therapy, the integration of moxibustion or warm needle acupuncture during the treatment of CSR significantly enhanced clinical response. Our research also demonstrated that the combination of moxibustion or warm needle acupuncture improves patient prognosis. This may be due to warm needle acupuncture or moxibustion can reduce local inflammatory factor activity and accelerate local tissue self-repair (31). Additionally, warm needle acupuncture can heat local muscles to promote blood circulation, improve local blood supply, and alleviate muscle spasms (32). Other studies (33, 34) showed that warm needle intervention improved the alignment of damaged muscle fibers and restored damaged large and small blood vessels. Mechanical compressive ischemia was alleviated under the heat induced by warm needle acupuncture, which can also increase 6-keto-PGF1α, reduce hs-CRP, IL-6, and TNF-α levels, increase the content of endothelial growth factors, promote local revascularization, and reduce inflammation (35). Our findings suggested that therapies combining warm needle acupuncture or moxibustion may yield superior clinical responses, which requires further investigation in clinical settings tailored to individual patient circumstances.

For pain relief, previous studies (17, 18, 20, 23) have shown that warm needle or fire needle acupuncture and needling therapy can more effectively alleviate pain, aligning with our results. In this study, we found that special and stimulating acupuncture methods exhibited greater efficacy in pain relief, potentially attributed to the additional reduction in cytokine levels, including tumor necrosis factor and IL-6 (24, 25), among patients receiving moxibustion and warm needle acupuncture adjunctive to standard acupuncture. This combined approach mitigated neuropathic pain, suppressed inflammatory responses, and alleviated pain symptoms. Dihui et al. (36) found that warm needle acupuncture can effectively reduce serum ET and malondialdehyde (MDA) levels, thereby alleviating pain symptoms. Studies (37, 38) demonstrated that tuina can relieve compressive cervicogenic pain caused by CSR, which corroborated our findings. This effect may be attributed to tuina’s effective reduction of serum substance P (SP) and prostaglandin E2 (PGE2) levels, both peripherally and centrally, thereby inhibiting pain transmission signals. Additionally, tuina may stimulate the periaqueductal gray (PAG) to release endogenous opioid peptides, consequently elevating the pain threshold and mitigating pain. Clinical and foundational studies on electroacupuncture (39–42) indicated that it can alleviate various types of pain associated with CSR. This effect may be due to electroacupuncture’s ability to reduce the expression of inflammatory markers such as Toll-like receptor 9 (TLR9), or modulate pain through the regulation of the P38MAPK and PI3K/Akt signaling pathways. Qi et al. (43) found that electroacupuncture can change the expression of genes in primary afferent neurons, reduce neurotransmitters involved in pain transmission, and regulate intraneuronal signaling pathways to inhibit the transmission of pain signals. Electroacupuncture can also suppress FOS protein and spinal dorsal horn cyclooxygenase-2 (COX-2) protein expression, thereby repairing damaged nerve roots (44), and alleviating pain symptoms. In addition, neurological studies (45, 46) indicated that electroacupuncture can enhance the pain threshold by modulating the spinal JAK2-STAT3-SOCS3 signaling pathway through the regulation of cannabinoid receptor CB1 and dopamine receptor D2. In treatments combining electroacupuncture and tuina, both methods can simultaneously relieve local muscle spasms and reduce mechanical compression on nerve roots. Junfeng et al. (47) found that rhythmic stimulation from electroacupuncture can cause cervical muscles to tremble, restoring biomechanical balance in the neck, reducing edema, lowering local tension, improving the state of compressed nerve roots, and then alleviating symptoms such as pain and numbness. Studies indicated that treatments combining electroacupuncture or massage appear to exhibit superior analgesic effects among all examined methods. However, to precisely evaluate the efficacy of these therapies, further in-depth analysis based on individual patient conditions is required.

Based on our study findings, no therapy has yet shown effectiveness in improving the physical signs of patients with CSR. A meta-analysis (48) demonstrated that implementing tuina did not statistically differ from not implementing tuina in terms of patient physical symptom scores, which is consistent with our findings. However, other research results, such as those reported by Jia et al. (49), suggested that tuina therapy was superior to cervical traction in improving symptoms and physical signs. This discrepancy may be attributed to heterogeneity among populations and differences in how treatment methods are applied. A meta-analysis (50) indicated that specific tuina techniques can lead to statistically significant improvements in cervical function compared to controls that did not employ these specific techniques. The report result is in line with our findings. This improvement may be attributed to manual techniques can restore the distribution of abnormal stresses on the cervical spine, enlarge the intervertebral foramen, reinstate the normal positional relationship between the spinous processes and the joints, correct cervical facet joint dysfunction, and restore anatomical alignment (51). Another meta-analysis (52) has shown that electroacupuncture was beneficial for cervical function, aligning with our results. This improvement may be attributed to the varying frequencies of electric currents used in electroacupuncture, which can increase the amplitude of muscle contractions. Consequently, this helps stretch the cervical vertebrae and reduce protrusions of intervertebral discs, while simultaneously improving the blood supply and nutrition to the cervical nerves and muscles (53). Additionally, clinical studies (54–58) have demonstrated that acupuncture or tuina and their combination therapies effectively enhanced cervical function and physical signs, and were recommended for combination therapy in clinical practice. The limited availability of direct comparative evidence resulted in sparse network connectivity for certain outcomes, particularly physical signs. This sparsity reduces the precision of indirect treatment effect estimates and warrants cautious interpretation of these findings. Future large-scale RCTs with standardized outcome measures are required to validate these preliminary findings.

To our knowledge, this is the first NMA that compares the effects of acupuncture or tuina combination therapy on pain, signs and symptoms, and clinical prognosis of patients with CSR. In this study, all therapies were ranked based on these factors. This study provided valuable information for determining the optimal treatment strategies for patients with CSR, thereby presenting the most efficacious clinical options. Nevertheless, several limitations in this NMA necessitate considerations. First, almost all included clinical studies did not adopt blinding, potentially associated with the administration methods of acupuncture. To mitigate this bias, methodological focus was placed on objectively measurable outcomes (specifically, VAS for pain, NDI for functional status, and the Spurling test for neurological signs) rather than subjective clinician evaluations. In accordance with strict PICOS criteria, studies with a high risk of bias (per RoB 2.0) in outcome measurement were excluded. Only RCTs with predefined protocols were included. These measures aimed to ensure the selection of high-quality RCTs, thereby enhancing the robustness and validity of the present analysis. However, potential biases remain and should be carefully considered when interpreting these findings. Future clinical trials should improve the methodological rigor of acupuncture delivery to further strengthen evidence quality. It is hoped that enhancements in the implementation of acupuncture in future clinical trials will mitigate adverse effects. Second, the small sample sizes in a substantial portion of clinical trials and the absence of sample size estimation could impact the accuracy and applicability of our findings. In addition, the limited direct evidence for certain indicators, such as physical signs, may reduce the robustness of indirect comparisons. Therefore, these results should be interpreted with caution. Furthermore, the subjective nature of treatment course selection, varying acupuncture frequencies, and inconsistent treatment numbers may lead to divergent cumulative effects. Significant heterogeneity across the included RCTs, particularly in sample sizes, intervention protocols (such as acupuncture techniques and treatment frequency), and follow-up durations, may limit the generalizability of our findings. Future studies with more rigorous designs are warranted. Moreover, under-reporting of key safety data was observed: only a small number of included RCTs recorded adverse events, precluding quantitative safety analyses. This lack of data hinders the risk–benefit assessments of invasive procedures such as acupuncture. Given the current lack of quantitative data, future research should prioritize systematic reviews focused on adverse event reporting. Lastly, the predominance of included studies from Chinese literature reflects a methodological challenge inherent in TCM research. As interventions such as acupuncture and tuina originated in China, the highest-quality RCTs are primarily conducted in this region. Despite comprehensive searches across four international databases (PubMed, Web of Science, Embase, and Cochrane Library) and inclusion of multinational populations, the disproportionate representation of Chinese studies remains unavoidable. Variations in genetic profiles, environmental exposures, and lifestyle factors across geographic populations may influence outcome interpretation. Consequently, our findings mainly apply to Asian populations, and extrapolation to other ethnic or regional groups should be made cautiously. To enhance external validity, future trials should include more diverse population cohorts and prioritize multinational collaborative research.

## Conclusion

Our study aimed to identify the most effective treatment methods, whether based on acupuncture, tuina therapy, or their combination therapies, for improving clinical response, pain, cervical function, and physical signs in CSR patients. The study found that acupuncture combined with warm needle acupuncture is most effective in improving clinical response in CSR patients, and electroacupuncture combined with moxibustion and conventional therapies provides the best outcomes in alleviating pain in CSR patients. Electroacupuncture combined with moxibustion and tuina, respectively, are most effective in improving symptoms and cervical function in CSR patients. Considering the constraints of current clinical research and evidence, future studies should prioritize larger sample sizes, extended follow-up durations, and more rigorous study designs to validate these findings.

## Data Availability

The original contributions presented in the study are included in the article/Supplementary material, further inquiries can be directed to the corresponding author.
