# Supplementary material for: Network meta-analysis of tuina or acupuncture in combination with adjunctive therapy for cervical spondylotic radiculopathy
Source: Front Neurol. 2025 Aug 8;16:1612024. doi: 10.3389/fneur.2025.1612024 (PMC12370718; doi:10.3389/fneur.2025.1612024)
Supplement: Supplementary file 2 [file Table_2.docx]

**Appendix 1:** Search strategies

**Pubmed**

**#1** "Radiculopathy"[Mesh]

**#2** ((((((((((((((((((((Radiculopathy[Title/Abstract]) OR (Radiculopathies[Title/Abstract])) OR (Cervical Radiculopathies[Title/Abstract])) OR (Nerve Root Disorder[Title/Abstract])) OR (Nerve Root Disorders[Title/Abstract])) OR (Radiculitis[Title/Abstract])) OR (Radiculitides[Title/Abstract])) OR (Nerve Root Inflammation[Title/Abstract])) OR (Nerve Root Inflammations[Title/Abstract])) OR (Nerve Root Avulsion[Title/Abstract])) OR (Avulsions, Nerve Root[Title/Abstract])) OR (Nerve Root Avulsions[Title/Abstract])) OR (Nerve Root Compression[Title/Abstract])) OR (Nerve Root Compressions[Title/Abstract])) OR (radiculopathy[Title/Abstract])) OR (nerve root disease[Title/Abstract])) OR (nerve root neuropathy[Title/Abstract])) OR (polyradiculopathy[Title/Abstract])) OR (radicular disease[Title/Abstract])) OR (radicular disorder[Title/Abstract])) OR (radicular neuropathy[Title/Abstract])

**#3** ("Radiculopathy"[Mesh]) OR (((((((((((((((((((((Radiculopathy[Title/Abstract]) OR (Radiculopathies[Title/Abstract])) OR (Cervical Radiculopathies[Title/Abstract])) OR (Nerve Root Disorder[Title/Abstract])) OR (Nerve Root Disorders[Title/Abstract])) OR (Radiculitis[Title/Abstract])) OR (Radiculitides[Title/Abstract])) OR (Nerve Root Inflammation[Title/Abstract])) OR (Nerve Root Inflammations[Title/Abstract])) OR (Nerve Root Avulsion[Title/Abstract])) OR (Avulsions, Nerve Root[Title/Abstract])) OR (Nerve Root Avulsions[Title/Abstract])) OR (Nerve Root Compression[Title/Abstract])) OR (Nerve Root Compressions[Title/Abstract])) OR (radiculopathy[Title/Abstract])) OR (nerve root disease[Title/Abstract])) OR (nerve root neuropathy[Title/Abstract])) OR (polyradiculopathy[Title/Abstract])) OR (radicular disease[Title/Abstract])) OR (radicular disorder[Title/Abstract])) OR (radicular neuropathy[Title/Abstract]))

**#4** "Spondylosis"[Mesh]

**#5** ((((((((((((Spondylosis[Title/Abstract]) OR (Lumbarsacral Spondylosis[Title/Abstract])) OR (Thoracic Spondylosis[Title/Abstract])) OR (Cervical Spondylosis[Title/Abstract])) OR (Spondylosis Deformans[Title/Abstract])) OR (spondylosis[Title/Abstract])) OR (arthrosis, spine[Title/Abstract])) OR (polyspondylitis marginalis osteophytica[Title/Abstract])) OR (spine arthrosis[Title/Abstract])) OR (spondylarthrosis[Title/Abstract])) OR (spondylo-arthrosis[Title/Abstract])) OR (spondyloarthrosis[Title/Abstract])) OR (spondyloses[Title/Abstract])

**#6** ("Spondylosis"[Mesh]) OR (((((((((((((Spondylosis[Title/Abstract]) OR (Lumbarsacral Spondylosis[Title/Abstract])) OR (Thoracic Spondylosis[Title/Abstract])) OR (Cervical Spondylosis[Title/Abstract])) OR (Spondylosis Deformans[Title/Abstract])) OR (spondylosis[Title/Abstract])) OR (arthrosis, spine[Title/Abstract])) OR (polyspondylitis marginalis osteophytica[Title/Abstract])) OR (spine arthrosis[Title/Abstract])) OR (spondylarthrosis[Title/Abstract])) OR (spondylo-arthrosis[Title/Abstract])) OR (spondyloarthrosis[Title/Abstract])) OR (spondyloses[Title/Abstract]))

**#7** (("Spondylosis"[Mesh]) OR (((((((((((((Spondylosis[Title/Abstract]) OR (Lumbarsacral Spondylosis[Title/Abstract])) OR (Thoracic Spondylosis[Title/Abstract])) OR (Cervical Spondylosis[Title/Abstract])) OR (Spondylosis Deformans[Title/Abstract])) OR (spondylosis[Title/Abstract])) OR (arthrosis, spine[Title/Abstract])) OR (polyspondylitis marginalis osteophytica[Title/Abstract])) OR (spine arthrosis[Title/Abstract])) OR (spondylarthrosis[Title/Abstract])) OR (spondylo-arthrosis[Title/Abstract])) OR (spondyloarthrosis[Title/Abstract])) OR (spondyloses[Title/Abstract]))) AND (("Radiculopathy"[Mesh]) OR (((((((((((((((((((((Radiculopathy[Title/Abstract]) OR (Radiculopathies[Title/Abstract])) OR (Cervical Radiculopathies[Title/Abstract])) OR (Nerve Root Disorder[Title/Abstract])) OR (Nerve Root Disorders[Title/Abstract])) OR (Radiculitis[Title/Abstract])) OR (Radiculitides[Title/Abstract])) OR (Nerve Root Inflammation[Title/Abstract])) OR (Nerve Root Inflammations[Title/Abstract])) OR (Nerve Root Avulsion[Title/Abstract])) OR (Avulsions, Nerve Root[Title/Abstract])) OR (Nerve Root Avulsions[Title/Abstract])) OR (Nerve Root Compression[Title/Abstract])) OR (Nerve Root Compressions[Title/Abstract])) OR (radiculopathy[Title/Abstract])) OR (nerve root disease[Title/Abstract])) OR (nerve root neuropathy[Title/Abstract])) OR (polyradiculopathy[Title/Abstract])) OR (radicular disease[Title/Abstract])) OR (radicular disorder[Title/Abstract])) OR (radicular neuropathy[Title/Abstract])))

**#8** (cervical spondylosis radiculopathy[Title/Abstract]) OR (Cervical spondylotic radiculopathy[Title/Abstract])

**#9**((cervical spondylosis radiculopathy[Title/Abstract]) OR (Cervical spondylotic radiculopathy[Title/Abstract])) OR ((("Spondylosis"[Mesh]) OR (((((((((((((Spondylosis[Title/Abstract]) OR (Lumbarsacral Spondylosis[Title/Abstract])) OR (Thoracic Spondylosis[Title/Abstract])) OR (Cervical Spondylosis[Title/Abstract])) OR (Spondylosis Deformans[Title/Abstract])) OR (spondylosis[Title/Abstract])) OR (arthrosis, spine[Title/Abstract])) OR (polyspondylitis marginalis osteophytica[Title/Abstract])) OR (spine arthrosis[Title/Abstract])) OR (spondylarthrosis[Title/Abstract])) OR (spondylo-arthrosis[Title/Abstract])) OR (spondyloarthrosis[Title/Abstract])) OR (spondyloses[Title/Abstract]))) AND (("Radiculopathy"[Mesh]) OR (((((((((((((((((((((Radiculopathy[Title/Abstract]) OR (Radiculopathies[Title/Abstract])) OR (Cervical Radiculopathies[Title/Abstract])) OR (Nerve Root Disorder[Title/Abstract])) OR (Nerve Root Disorders[Title/Abstract])) OR (Radiculitis[Title/Abstract])) OR (Radiculitides[Title/Abstract])) OR (Nerve Root Inflammation[Title/Abstract])) OR (Nerve Root Inflammations[Title/Abstract])) OR (Nerve Root Avulsion[Title/Abstract])) OR (Avulsions, Nerve Root[Title/Abstract])) OR (Nerve Root Avulsions[Title/Abstract])) OR (Nerve Root Compression[Title/Abstract])) OR (Nerve Root Compressions[Title/Abstract])) OR (radiculopathy[Title/Abstract])) OR (nerve root disease[Title/Abstract])) OR (nerve root neuropathy[Title/Abstract])) OR (polyradiculopathy[Title/Abstract])) OR (radicular disease[Title/Abstract])) OR (radicular disorder[Title/Abstract])) OR (radicular neuropathy[Title/Abstract]))))

**#10** "Acupuncture"[Mesh] OR "Acupuncture Therapy"[Mesh] OR "Acupuncture, Ear"[Mesh] OR "Acupuncture Points"[Mesh]

**#11** "Massage"[Mesh]

**#12** ("Massage"[Mesh]) OR ("Acupuncture"[Mesh] OR "Acupuncture Therapy"[Mesh] OR "Acupuncture, Ear"[Mesh] OR "Acupuncture Points"[Mesh])

**#13**((((((((((((((((((((((((((((((((((((((((((((((((((((((Acupuncture[Title/Abstract]) OR (Pharmacopuncture[Title/Abstract])) OR (Meridian[Title/Abstract])) OR (acupuncture[Title/Abstract])) OR (shonishin[Title/Abstract])) OR (acupuncture therapy[Title/Abstract])) OR (Acupuncture Treatment[Title/Abstract])) OR (Acupuncture Treatments[Title/Abstract])) OR (Pharmacoacupuncture Treatment[Title/Abstract])) OR (Pharmacoacupuncture Therapy[Title/Abstract])) OR (Acupotomy[Title/Abstract])) OR (Acupotomies[Title/Abstract])) OR (Acupuncture, Ear[Title/Abstract])) OR (Ear Acupunctures[Title/Abstract])) OR (Auricular Acupuncture[Title/Abstract])) OR (Ear Acupuncture[Title/Abstract])) OR (Auricular Acupunctures[Title/Abstract])) OR (auricular acupuncture[Title/Abstract])) OR (auriculo-acupuncture[Title/Abstract])) OR (auriculoacupuncture[Title/Abstract])) OR (auriculotherapy[Title/Abstract])) OR (electroacupuncture[Title/Abstract])) OR (electroacupuncture[Title/Abstract])) OR (acupuncture, electric[Title/Abstract])) OR (electric acupuncture[Title/Abstract])) OR (electrical acupoint stimulation[Title/Abstract])) OR (electrical acupuncture[Title/Abstract])) OR (electro-acupuncture[Title/Abstract])) OR (electrode acupuncture[Title/Abstract])) OR (electronic acupuncture[Title/Abstract])) OR (acupuncture points[Title/Abstract])) OR (acupuncture point[Title/Abstract])) OR (Acupoints[Title/Abstract])) OR (Acupoint[Title/Abstract])) OR (acu-point[Title/Abstract])) OR (Warm needling[Title/Abstract])) OR (Fire needling[Title/Abstract])) OR (Auriculotherapy[Title/Abstract])) OR (dry needling[Title/Abstract])) OR (shonishin[Title/Abstract])) OR (electrical acupoint stimulation[Title/Abstract])) OR (Massage[Title/Abstract])) OR (Zone Therapy[Title/Abstract])) OR (Zone Therapies[Title/Abstract])) OR (Massage Therapy[Title/Abstract])) OR (Massage Therapies[Title/Abstract])) OR (masso-therapy[Title/Abstract])) OR (massotherapy[Title/Abstract])) OR (Tuina[Title/Abstract])) OR (Tui na[Title/Abstract])) OR (Anmo[Title/Abstract])) OR (An mo[Title/Abstract])) OR (manipulation[Title/Abstract])) OR (naprapathy[Title/Abstract])) OR (manipulative therapeutics[Title/Abstract])

**#14** (("Massage"[Mesh]) OR ("Acupuncture"[Mesh] OR "Acupuncture Therapy"[Mesh] OR "Acupuncture, Ear"[Mesh] OR "Acupuncture Points"[Mesh])) OR (((((((((((((((((((((((((((((((((((((((((((((((((((((((Acupuncture[Title/Abstract]) OR (Pharmacopuncture[Title/Abstract])) OR (Meridian[Title/Abstract])) OR (acupuncture[Title/Abstract])) OR (shonishin[Title/Abstract])) OR (acupuncture therapy[Title/Abstract])) OR (Acupuncture Treatment[Title/Abstract])) OR (Acupuncture Treatments[Title/Abstract])) OR (Pharmacoacupuncture Treatment[Title/Abstract])) OR (Pharmacoacupuncture Therapy[Title/Abstract])) OR (Acupotomy[Title/Abstract])) OR (Acupotomies[Title/Abstract])) OR (Acupuncture, Ear[Title/Abstract])) OR (Ear Acupunctures[Title/Abstract])) OR (Auricular Acupuncture[Title/Abstract])) OR (Ear Acupuncture[Title/Abstract])) OR (Auricular Acupunctures[Title/Abstract])) OR (auricular acupuncture[Title/Abstract])) OR (auriculo-acupuncture[Title/Abstract])) OR (auriculoacupuncture[Title/Abstract])) OR (auriculotherapy[Title/Abstract])) OR (electroacupuncture[Title/Abstract])) OR (electroacupuncture[Title/Abstract])) OR (acupuncture, electric[Title/Abstract])) OR (electric acupuncture[Title/Abstract])) OR (electrical acupoint stimulation[Title/Abstract])) OR (electrical acupuncture[Title/Abstract])) OR (electro-acupuncture[Title/Abstract])) OR (electrode acupuncture[Title/Abstract])) OR (electronic acupuncture[Title/Abstract])) OR (acupuncture points[Title/Abstract])) OR (acupuncture point[Title/Abstract])) OR (Acupoints[Title/Abstract])) OR (Acupoint[Title/Abstract])) OR (acu-point[Title/Abstract])) OR (Warm needling[Title/Abstract])) OR (Fire needling[Title/Abstract])) OR (Auriculotherapy[Title/Abstract])) OR (dry needling[Title/Abstract])) OR (shonishin[Title/Abstract])) OR (electrical acupoint stimulation[Title/Abstract])) OR (Massage[Title/Abstract])) OR (Zone Therapy[Title/Abstract])) OR (Zone Therapies[Title/Abstract])) OR (Massage Therapy[Title/Abstract])) OR (Massage Therapies[Title/Abstract])) OR (masso-therapy[Title/Abstract])) OR (massotherapy[Title/Abstract])) OR (Tuina[Title/Abstract])) OR (Tui na[Title/Abstract])) OR (Anmo[Title/Abstract])) OR (An mo[Title/Abstract])) OR (manipulation[Title/Abstract])) OR (naprapathy[Title/Abstract])) OR (manipulative therapeutics[Title/Abstract]))

**#15** ((("Massage"[Mesh]) OR ("Acupuncture"[Mesh] OR "Acupuncture Therapy"[Mesh] OR "Acupuncture, Ear"[Mesh] OR "Acupuncture Points"[Mesh])) OR (((((((((((((((((((((((((((((((((((((((((((((((((((((((Acupuncture[Title/Abstract]) OR (Pharmacopuncture[Title/Abstract])) OR (Meridian[Title/Abstract])) OR (acupuncture[Title/Abstract])) OR (shonishin[Title/Abstract])) OR (acupuncture therapy[Title/Abstract])) OR (Acupuncture Treatment[Title/Abstract])) OR (Acupuncture Treatments[Title/Abstract])) OR (Pharmacoacupuncture Treatment[Title/Abstract])) OR (Pharmacoacupuncture Therapy[Title/Abstract])) OR (Acupotomy[Title/Abstract])) OR (Acupotomies[Title/Abstract])) OR (Acupuncture, Ear[Title/Abstract])) OR (Ear Acupunctures[Title/Abstract])) OR (Auricular Acupuncture[Title/Abstract])) OR (Ear Acupuncture[Title/Abstract])) OR (Auricular Acupunctures[Title/Abstract])) OR (auricular acupuncture[Title/Abstract])) OR (auriculo-acupuncture[Title/Abstract])) OR (auriculoacupuncture[Title/Abstract])) OR (auriculotherapy[Title/Abstract])) OR (electroacupuncture[Title/Abstract])) OR (electroacupuncture[Title/Abstract])) OR (acupuncture, electric[Title/Abstract])) OR (electric acupuncture[Title/Abstract])) OR (electrical acupoint stimulation[Title/Abstract])) OR (electrical acupuncture[Title/Abstract])) OR (electro-acupuncture[Title/Abstract])) OR (electrode acupuncture[Title/Abstract])) OR (electronic acupuncture[Title/Abstract])) OR (acupuncture points[Title/Abstract])) OR (acupuncture point[Title/Abstract])) OR (Acupoints[Title/Abstract])) OR (Acupoint[Title/Abstract])) OR (acu-point[Title/Abstract])) OR (Warm needling[Title/Abstract])) OR (Fire needling[Title/Abstract])) OR (Auriculotherapy[Title/Abstract])) OR (dry needling[Title/Abstract])) OR (shonishin[Title/Abstract])) OR (electrical acupoint stimulation[Title/Abstract])) OR (Massage[Title/Abstract])) OR (Zone Therapy[Title/Abstract])) OR (Zone Therapies[Title/Abstract])) OR (Massage Therapy[Title/Abstract])) OR (Massage Therapies[Title/Abstract])) OR (masso-therapy[Title/Abstract])) OR (massotherapy[Title/Abstract])) OR (Tuina[Title/Abstract])) OR (Tui na[Title/Abstract])) OR (Anmo[Title/Abstract])) OR (An mo[Title/Abstract])) OR (manipulation[Title/Abstract])) OR (naprapathy[Title/Abstract])) OR (manipulative therapeutics[Title/Abstract]))) AND (((cervical spondylosis radiculopathy[Title/Abstract]) OR (Cervical spondylotic radiculopathy[Title/Abstract])) OR ((("Spondylosis"[Mesh]) OR (((((((((((((Spondylosis[Title/Abstract]) OR (Lumbarsacral Spondylosis[Title/Abstract])) OR (Thoracic Spondylosis[Title/Abstract])) OR (Cervical Spondylosis[Title/Abstract])) OR (Spondylosis Deformans[Title/Abstract])) OR (spondylosis[Title/Abstract])) OR (arthrosis, spine[Title/Abstract])) OR (polyspondylitis marginalis osteophytica[Title/Abstract])) OR (spine arthrosis[Title/Abstract])) OR (spondylarthrosis[Title/Abstract])) OR (spondylo-arthrosis[Title/Abstract])) OR (spondyloarthrosis[Title/Abstract])) OR (spondyloses[Title/Abstract]))) AND (("Radiculopathy"[Mesh]) OR (((((((((((((((((((((Radiculopathy[Title/Abstract]) OR (Radiculopathies[Title/Abstract])) OR (Cervical Radiculopathies[Title/Abstract])) OR (Nerve Root Disorder[Title/Abstract])) OR (Nerve Root Disorders[Title/Abstract])) OR (Radiculitis[Title/Abstract])) OR (Radiculitides[Title/Abstract])) OR (Nerve Root Inflammation[Title/Abstract])) OR (Nerve Root Inflammations[Title/Abstract])) OR (Nerve Root Avulsion[Title/Abstract])) OR (Avulsions, Nerve Root[Title/Abstract])) OR (Nerve Root Avulsions[Title/Abstract])) OR (Nerve Root Compression[Title/Abstract])) OR (Nerve Root Compressions[Title/Abstract])) OR (radiculopathy[Title/Abstract])) OR (nerve root disease[Title/Abstract])) OR (nerve root neuropathy[Title/Abstract])) OR (polyradiculopathy[Title/Abstract])) OR (radicular disease[Title/Abstract])) OR (radicular disorder[Title/Abstract])) OR (radicular neuropathy[Title/Abstract])))))

**Embase**

**#1** 'radiculopathy'/exp

**#2** radiculopathies:ti,ab,kw OR 'cervical radiculopathies':ti,ab,kw OR 'nerve root disorder':ti,ab,kw OR 'nerve root disorders':ti,ab,kw OR radiculitis:ti,ab,kw OR radiculitides:ti,ab,kw OR 'nerve root inflammation':ti,ab,kw OR 'nerve root inflammations':ti,ab,kw OR 'nerve root avulsion':ti,ab,kw OR 'avulsions, nerve root':ti,ab,kw OR 'nerve root avulsions':ti,ab,kw OR 'nerve root compression':ti,ab,kw OR 'nerve root compressions':ti,ab,kw OR radiculopathy:ti,ab,kw OR 'nerve root disease':ti,ab,kw OR 'nerve root neuropathy':ti,ab,kw OR polyradiculopathy:ti,ab,kw OR 'radicular disease':ti,ab,kw OR 'radicular disorder':ti,ab,kw OR 'radicular neuropathy':ti,ab,kw

**#3** 'spondylosis'/exp

**#4** 'lumbarsacral spondylosis':ti,ab,kw OR 'thoracic spondylosis':ti,ab,kw OR 'cervical spondylosis':ti,ab,kw OR 'spondylosis deformans':ti,ab,kw OR spondylosis:ti,ab,kw OR 'arthrosis, spine':ti,ab,kw OR 'polyspondylitis marginalis osteophytica':ti,ab,kw OR 'spine arthrosis':ti,ab,kw OR spondylarthrosis:ti,ab,kw OR 'spondylo arthrosis':ti,ab,kw OR spondyloarthrosis:ti,ab,kw OR spondyloses:ti,ab,kw

**#5** cervical spondylosis radiculopathy':ti,ab,kw OR 'cervical spondylotic radiculopathy':ti,ab,kw

**#6** 1 OR #2

**#7** 3 OR #4

**#8** #6 AND #7

**#9** #5 OR #8

**#10** 'acupuncture'/exp OR 'auricular acupuncture'/exp OR 'electroacupuncture'/exp OR 'acupuncture point'/exp OR 'massage'/exp

**#11** pharmacopuncture:ti,ab,kw OR meridian:ti,ab,kw OR acupuncture:ti,ab,kw OR 'acupuncture treatment':ti,ab,kw OR 'acupuncture therapy':ti,ab,kw OR 'acupuncture treatments':ti,ab,kw OR 'pharmacoacupuncture treatment':ti,ab,kw OR 'pharmacoacupuncture therapy':ti,ab,kw OR acupotomy:ti,ab,kw OR acupotomies:ti,ab,kw OR 'acupuncture, ear':ti,ab,kw OR 'ear acupunctures':ti,ab,kw OR 'ear acupuncture':ti,ab,kw OR 'auricular acupunctures':ti,ab,kw OR 'auricular acupuncture':ti,ab,kw OR 'auriculo acupuncture':ti,ab,kw OR auriculoacupuncture:ti,ab,kw OR electroacupuncture:ti,ab,kw OR 'acupuncture, electric':ti,ab,kw OR 'electric acupuncture':ti,ab,kw OR 'electrical acupuncture':ti,ab,kw OR 'electro acupuncture':ti,ab,kw OR 'electrode acupuncture':ti,ab,kw OR 'electronic acupuncture':ti,ab,kw OR 'acupuncture points':ti,ab,kw OR 'acupuncture point':ti,ab,kw OR acupoints:ti,ab,kw OR acupoint:ti,ab,kw OR 'acu point':ti,ab,kw OR 'warm needling':ti,ab,kw OR 'fire needling':ti,ab,kw OR auriculotherapy:ti,ab,kw OR 'dry needling':ti,ab,kw OR shonishin:ti,ab,kw OR 'electrical acupoint stimulation':ti,ab,kw OR massage:ti,ab,kw OR 'zone therapy':ti,ab,kw OR 'zone therapies':ti,ab,kw OR 'massage therapy':ti,ab,kw OR 'massage therapies':ti,ab,kw OR 'masso therapy':ti,ab,kw OR massotherapy:ti,ab,kw OR tuina:ti,ab,kw OR 'tui na':ti,ab,kw OR anmo:ti,ab,kw OR 'an mo':ti,ab,kw OR manipulation:ti,ab,kw OR naprapathy:ti,ab,kw OR 'manipulative therapeutics':ti,ab,kw

**#12** #10 OR #11

**#13** 9 AND #12

**Cochrane Library**

**#1** MeSH descriptor: [Radiculopathy] explode all trees

**#2** (Radiculopathy):ti,ab,kw OR (Radiculopathies):ti,ab,kw OR (Cervical Radiculopathies):ti,ab,kw OR (Nerve Root Disorder):ti,ab,kw OR (Nerve Root Disorders):ti,ab,kw

**#3** (Radiculitis):ti,ab,kw OR (Radiculitides):ti,ab,kw OR (Nerve Root Inflammation):ti,ab,kw OR (Nerve Root Inflammations):ti,ab,kw OR (Nerve Root Avulsion):ti,ab,kw

**#4** (Avulsions, Nerve Root):ti,ab,kw OR (Nerve Root Avulsions):ti,ab,kw OR (Nerve Root Compression):ti,ab,kw OR (Compressions, Nerve Root):ti,ab,kw OR (Nerve Root Compressions):ti,ab,kw

**#5** (radiculopathy):ti,ab,kw OR (nerve root disease):ti,ab,kw OR (nerve root neuropathy):ti,ab,kw OR (polyradiculopathy):ti,ab,kw OR (radicular disease):ti,ab,kw

**#6** (radicular disorder):ti,ab,kw OR (radicular neuropathy):ti,ab,kw

**#7** #1 or #2 or #3 or #4 or #5 or #6

**#8** MeSH descriptor: [Spondylosis] explode all trees

**#9** (Spondylosis):ti,ab,kw OR (Lumbarsacral Spondylosis):ti,ab,kw OR (Thoracic Spondylosis):ti,ab,kw OR (Cervical Spondylosis):ti,ab,kw OR (Spondylosis Deformans):ti,ab,kw

**#10** (arthrosis, spine):ti,ab,kw OR (polyspondylitis marginalis osteophytica):ti,ab,kw OR (spine arthrosis):ti,ab,kw OR (spondylarthrosis):ti,ab,kw OR (spondylo-arthrosis):ti,ab,kw

**#11** (spondyloarthrosis):ti,ab,kw OR (spondyloses):ti,ab,kw

**#12** 8 or #9 or #10 or #11

**#13** (cervical spondylosis radiculopathy):ti,ab,kw OR (Cervical spondylotic radiculopathy):ti,ab,kw

**#14** 12 and #7

**#15** #13 or #14

**#16** MeSH descriptor: [Acupuncture] explode all trees

**#17** MeSH descriptor: [Acupuncture Therapy] explode all trees

**#18** MeSH descriptor: [Acupuncture, Ear] explode all trees

**#19** MeSH descriptor: [Electroacupuncture] explode all trees

**#20** MeSH descriptor: [Acupuncture Points] explode all trees

**#21** MeSH descriptor: [Massage] explode all trees

**#22** #16 or #17 or #18 or #19 or #20 or #21

**#23** (Acupuncture):ti,ab,kw OR (Pharmacopuncture):ti,ab,kw OR (Meridian):ti,ab,kw OR (acupuncture):ti,ab,kw OR (shonishin):ti,ab,kw

**#24** (acupuncture treatment):ti,ab,kw OR (acupuncture therapy):ti,ab,kw OR (Acupuncture Treatments):ti,ab,kw OR (Pharmacoacupuncture Treatment):ti,ab,kw OR (Pharmacoacupuncture Therapy):ti,ab,kw

**#25** (Acupotomy):ti,ab,kw OR (Acupotomies):ti,ab,kw OR (Acupuncture, Ear):ti,ab,kw OR (Ear Acupunctures):ti,ab,kw OR (Auricular Acupuncture):ti,ab,kw

**#26** (Ear Acupuncture):ti,ab,kw OR (Auricular Acupunctures):ti,ab,kw OR (auricular acupuncture):ti,ab,kw OR (auriculo-acupuncture):ti,ab,kw OR (auriculoacupuncture):ti,ab,kw

**#27** (auriculotherapy):ti,ab,kw OR (earlobe acupuncture):ti,ab,kw OR (electroacupuncture):ti,ab,kw OR (acupuncture, electric):ti,ab,kw OR (electric acupuncture):ti,ab,kw

**#28** (electrical acupoint stimulation):ti,ab,kw OR (electrical acupuncture):ti,ab,kw OR (electro-acupuncture):ti,ab,kw OR (electrode acupuncture):ti,ab,kw OR (electronic acupuncture):ti,ab,kw

**#29** (acupuncture points):ti,ab,kw OR (acupuncture point):ti,ab,kw OR (Acupoints):ti,ab,kw OR (Acupoint):ti,ab,kw OR (acu-point):ti,ab,kw

**#30** (Scalp acupuncture):ti,ab,kw OR (Ocular acupuncture):ti,ab,kw OR (Warm needling):ti,ab,kw OR (Fire needling):ti,ab,kw OR (Auriculotherapy):ti,ab,kw

**#31** (dry needling):ti,ab,kw OR (shonishin):ti,ab,kw OR (electrical acupoint stimulation):ti,ab,kw OR (electric acupuncture):ti,ab,kw OR (Massage):ti,ab,kw

**#32** (Zone Therapy):ti,ab,kw OR (Zone Therapies):ti,ab,kw OR (Massage Therapy):ti,ab,kw OR (Massage Therapies):ti,ab,kw OR (masso-therapy):ti,ab,kw

**#33** (Therapies, Massage):ti,ab,kw OR (massotherapy):ti,ab,kw OR (Tuina):ti,ab,kw OR (Tui na):ti,ab,kw OR (Anmo):ti,ab,kw

**#34** (An mo):ti,ab,kw OR (manipulation):ti,ab,kw OR (naprapathy):ti,ab,kw OR (manipulative therapeutics):ti,ab,kw

**#35** #23 or #24 or #25 or #26 or #27 or #28 or #29 or #30 or #31 or #32 or #33 or #34

**#36** #35 or #22

**#37** #15 and #36

**Web of Science**

**#1** Radiculopathy (Topic) or Radiculopathies (Topic) or Cervical Radiculopathies (Topic) or Nerve Root Disorder (Topic) or Nerve Root Disorders (Topic) or Radiculitis (Topic) or Radiculitides (Topic) or Nerve Root Inflammation (Topic) or Nerve Root Inflammations (Topic) or Nerve Root Avulsion (Topic) or Nerve Root Avulsions (Topic) or Nerve Root Compression (Topic) or Nerve Root Compressions (Topic) or radiculopathy (Topic) or nerve root disease (Topic) or nerve root neuropathy (Topic) or polyradiculopathy (Topic) or radicular disease (Topic) or radicular disorder (Topic) or radicular neuropathy (Topic)

**#2** pondylosis (Topic) or Lumbarsacral Spondylosis (Topic) or Thoracic Spondylosis (Topic) or Cervical Spondylosis (Topic) or Spondylosis Deformans (Topic) or spondylosis (Topic) or arthrosis, spine (Topic) or polyspondylitis marginalis osteophytica (Topic) or spine arthrosis (Topic) or spondylarthrosis (Topic) or spondylo-arthrosis (Topic) or spondyloarthrosis (Topic) or spondyloses (Topic)

**#3** cupuncture (Topic) or Pharmacopuncture (Topic) or Meridian (Topic) or acupuncture (Topic) or shonishin (Topic) or acupuncture therapy (Topic) or Acupuncture Treatment (Topic) or Acupuncture Treatments (Topic) or Pharmacoacupuncture Treatment (Topic) or Pharmacoacupuncture Therapy (Topic) or Acupotomy (Topic) or Acupotomies (Topic) or Acupuncture, Ear (Topic) or Ear Acupunctures (Topic) or Auricular Acupuncture (Topic) or Ear Acupuncture (Topic) or Auricular Acupunctures (Topic) or auricular acupuncture (Topic) or auriculo-acupuncture (Topic) or auriculoacupuncture (Topic) or auriculotherapy (Topic) or earlobe acupuncture (Topic) or electroacupuncture (Topic) or acupuncture, electric (Topic) or electric acupuncture (Topic) or electrical acupoint stimulation (Topic) or electrical acupuncture (Topic) or electro-acupuncture (Topic) or electrode acupuncture (Topic) or electronic acupuncture (Topic) or acupuncture points (Topic) or acupuncture point (Topic) or Acupoints (Topic) or Acupoint (Topic) or acu-point (Topic) or Scalp acupuncture (Topic) or Ocular acupuncture (Topic) or Warm needling (Topic) or Fire needling (Topic) or dry needling (Topic) or shonishin (Topic) or electrical acupoint stimulation (Topic) or Massage (Topic) or Zone Therapy (Topic) or Zone Therapies (Topic) or Massage Therapy (Topic) or Massage Therapies (Topic) or Embase ; Massage (Topic) or masso-therapy (Topic) or massotherapy (Topic) or Tuina (Topic) or Tui na (Topic) or Anmo (Topic) or An mo (Topic) or manipulation (Topic) or naprapathy (Topic) or manipulative therapeutics (Topic)

**#4** ervical spondylosis radiculopathy (Topic) or Cervical spondylotic radiculopathy (Topic)

**#5** #1 AND #2

**#6** #5 OR #4

**#7** #3 AND #6

**Note: search in Chines for Chinese database**

**VIP**
((((((((((((((((((((((摘要=针灸 OR 摘要=温针灸) OR 摘要=电针) OR 摘要=针刺) OR 摘要=耳针) OR 摘要=头针) OR 摘要=头皮针) OR 摘要=温和灸) OR 摘要=温针疗法) OR 摘要=耳廓针刺术) OR 摘要=耳埋) OR 摘要=按摩) OR 摘要=推拿) AND ((摘要=神经根型颈椎病 OR 摘要=神经根性颈椎病) OR ((摘要=神经根性 OR 摘要=神经根型) AND 摘要=颈椎病))))))) AND ((摘要=临床研究 OR 摘要=随机对照) OR 摘要=临床试验))))))

**CNKI**
TKA = ('针灸'+'温针灸'+'电针'+'针刺'+'耳针'+'头针'+'头皮针'+'温和灸'+'温针疗法'+'耳廓针刺术'+'耳埋'+'按摩'+'推拿') AND TKA = ('神经根型颈椎病'+'神经根性颈椎病') AND TKA = ('临床研究'+'随机对照')

**Wanfang**
主题:(针灸 or 温针灸 or 电针 or 针刺 or 耳针 or 头针 or 头皮针 or 温和灸 or 温针疗法 or 耳廓针刺术 or 耳埋 or 按摩 or 推拿) and 主题:(神经根型颈椎病 or 神经根性颈椎病) and 主题:(临床研究 or 随机对照) 713

**CBM**

("针灸疗法"[不加权:扩展]) OR "肌肉骨骼手法"[不加权:扩展] OR "推拿疗法"[不加权:扩展]

"针灸疗法"[常用字段:智能] OR "温针灸"[常用字段:智能] OR "电针"[常用字段:智能] OR "耳针"[常用字段:智能] OR "头针"[常用字段:智能] OR "针刺"[常用字段:智能] OR "推拿"[常用字段:智能] OR "按摩"[常用字段:智能] OR "耳廓针刺术"[常用字段:智能]

(#2) OR (#1)

"神经根型颈椎病"[常用字段:智能] OR "神经根性颈椎病"[常用字段:智能]

"临床实验"[常用字段:智能] OR "临床试验"[常用字段:智能] OR "随机对照"[常用字段:智能]

(#5) AND (#4) AND (#3)
