# Supplementary material for: Network meta-analysis of tuina or acupuncture in combination with adjunctive therapy for cervical spondylotic radiculopathy
Source: Front Neurol. 2025 Aug 8;16:1612024. doi: 10.3389/fneur.2025.1612024 (PMC12370718; doi:10.3389/fneur.2025.1612024)
Supplement: Supplementary file 5 [file Table_5.docx]

**Appendix 5:**

**Figure S1** Publication bias of Clinical response-testing funnel plots

**Figure S2** Publication bias test funnel plot for pain

**Figure S3** Publication bias funnel plot for Cervical spine function

**Figure S4** Funnel plots for publication bias testing of Physical signs

**
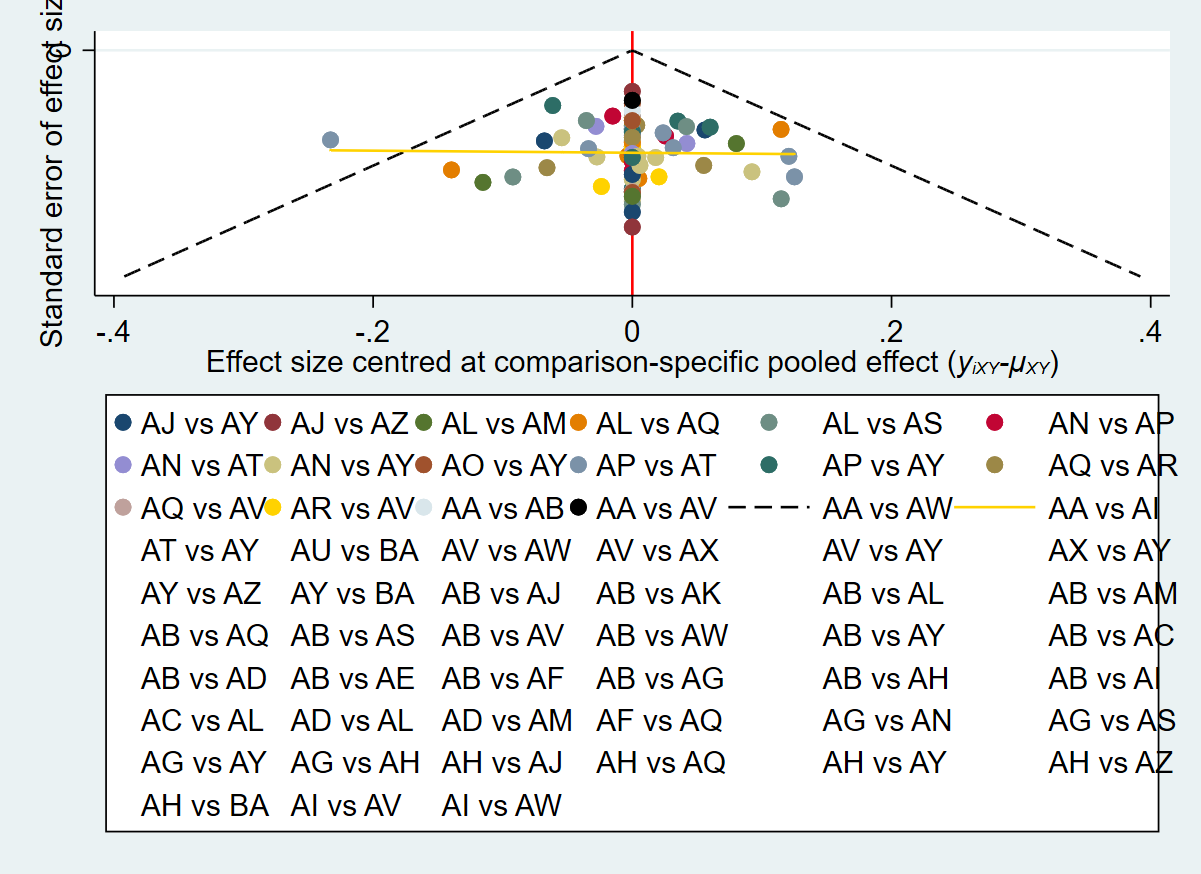
**

**Figure S1** Publication bias of Clinical response-testing funnel plots

**
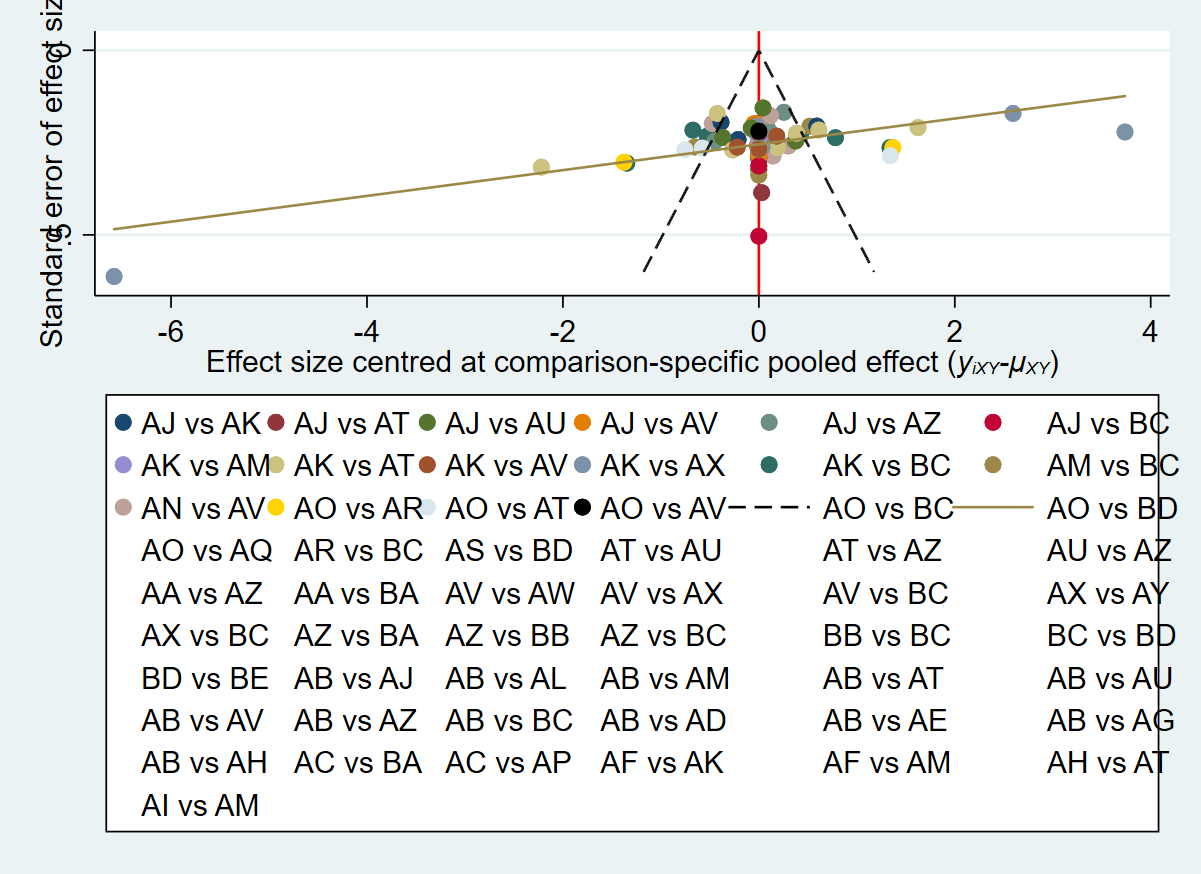
Figure S2** Publication bias test funnel plot for pain

**
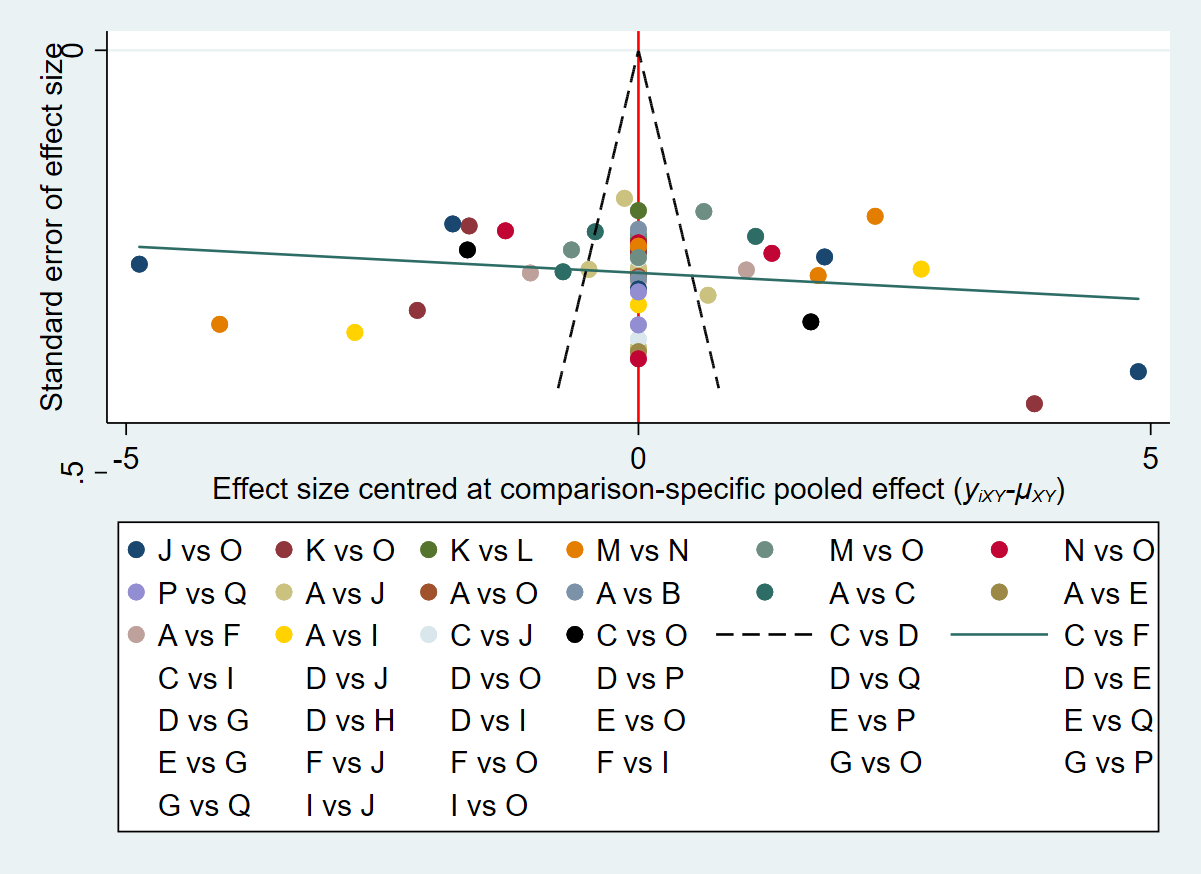
Figure S3** Publication bias funnel plot for Cervical spine function

**
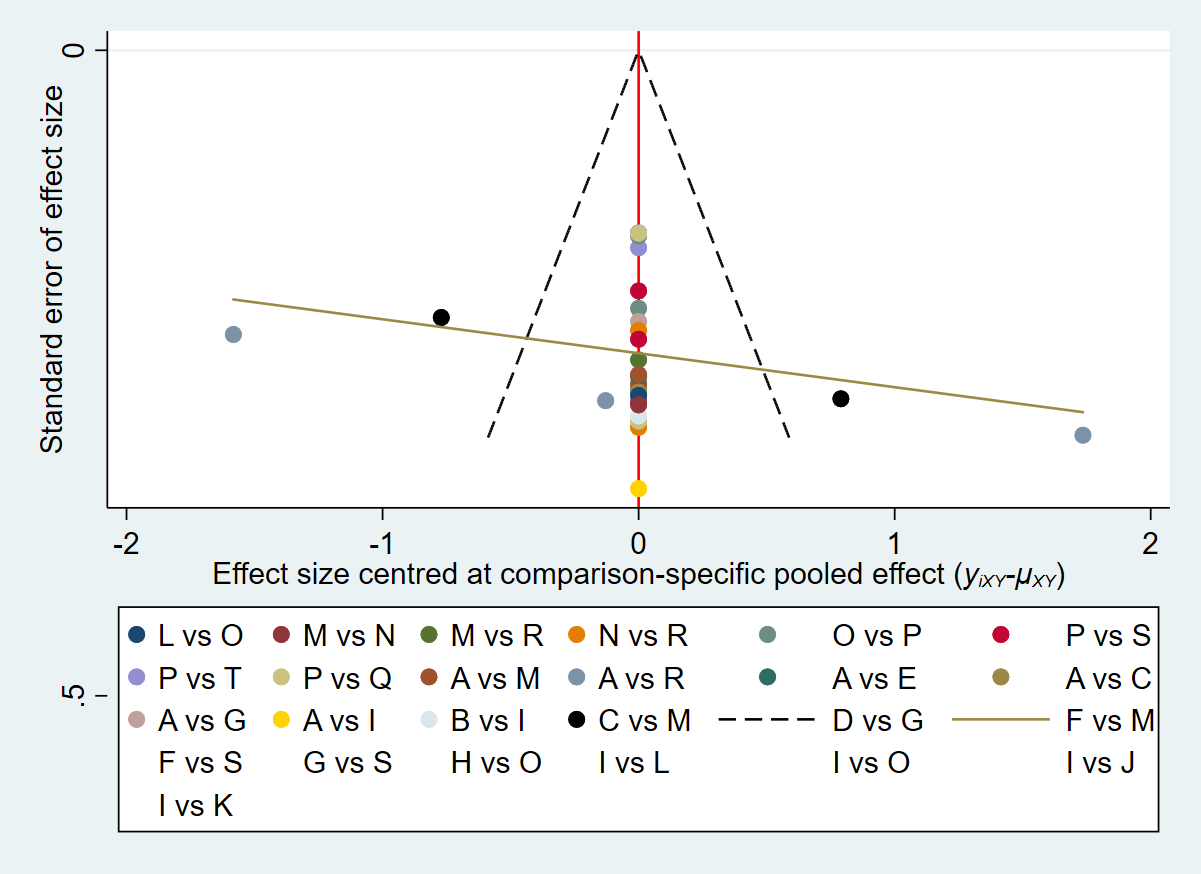
Figure S4** Funnel plots for publication bias testing of Physical signs
