# Supplementary material for: Network meta-analysis of tuina or acupuncture in combination with adjunctive therapy for cervical spondylotic radiculopathy
Source: Front Neurol. 2025 Aug 8;16:1612024. doi: 10.3389/fneur.2025.1612024 (PMC12370718; doi:10.3389/fneur.2025.1612024)
Supplement: Supplementary file 6 [file Table_6.docx]

**Appendix 3:** References of included studies in the NMA

[1] B. Li, J. Li, Effect of acupuncture on surface electromyographic signals of cervical muscles in patients with cervical spondylotic radiculopathy, Chinese Journal of Physical Medicine and Rehabilitation 35(5) (2013) 395-397. DOI: 10.3760/cma.j.issn.0254-1424.2013.05.018

[2] Z. Mi, J. Bi, Clinical observation on the therapeutic effect of massage on cervical spondylotic radiculopathy, Western Journal of Traditional Chinese Medicine 26(7) (2013) 101-102. DOI: 10.3969/j.issn.1004-6852.2013.07.038

[3] YuliangWang, Z. Wang, Observation on the therapeutic effect of acupuncture and moxibustion on cervical spondylotic radiculopathy, Liaoning Journal of Traditional Chinese Medicine 40(11) (2013) 2342-2344. DOI: 10.13192/j.issn.1000-1719.2013.11.004

[4] H. Xie, N. Wen, B. Zheng, Y. Li, Clinical observation on acupuncture combined with Bianstone therapy for the treatment of cervical spondylotic radiculopathy, Journal of Clinical Acupuncture and Moxibustion 29(5) (2013) 1-4. DOI: 10.3969/j.issn.1005-0779.2013.05.001

[5] X. Bu, J. Sun, Clinical observation on acupuncture combined with radiofrequency ablation in the treatment of cervical spondylotic radiculopathy, Western Journal of Traditional Chinese Medicine (7) (2014) 99-101.

[6] Y. Wang, W. Shen, W. Wang, Evaluation of the quality of life of patients with cervical spondylotic radiculopathy treated with "eight-needle neck", Liaoning Journal of Traditional Chinese Medicine 41(6) (2014) 1254-1256. DOI: 10.13192/j.issn.1000-1719.2014.06.078

[7] Y. Wang, W. Shen, W. Wang, H. Zhang, Evaluation of the effect of "eight-needle acupuncture in the neck" on pain of cervical spondylotic radiculopathy using simplified McGill scale, Journal of Clinical Acupuncture and Moxibustion (1) (2014) 7-10. DOI: 10.3969/j.issn.1005-0779.2014.01.003

[8] K. Wu, Observation on the therapeutic effect of auricular acupoint pressing combined with electroacupuncture in the treatment of cervical spondylotic radiculopathy, Journal of Emergency in Traditional Chinese Medicine 23(7) (2014) 1360-1361. DOI: 10.3969/j.issn.1004-745X.2014.07.065

[9] F. Zhao, C. Wang, Y. Zhang, Q. Zhou, Massage combined with health exercises to treat cervical spondylosis of cervical type and radiculopathy, Jilin Journal of Traditional Chinese Medicine 34(9) (2014) 951-955. DOI: 10.13463/j.cnki.jlzyy.2014.09.026

[10] Z. Feng, L. Li, Observation on the therapeutic effect of comprehensive rehabilitation therapy on cervical spondylotic radiculopathy, Shandong Journal of Traditional Chinese Medicine 34(11) (2015) 844-846,873.

[11] F. Gao, J. Zhu, X. Yang, Y. Yao, Observation on the therapeutic effect of comprehensive rehabilitation therapy on cervical spondylotic radiculopathy, Chinese Journal of General Practice 7(13) (2015) 1174-1175.

[12] X. Wang, H. Zhan, M. Zhang, Y. Chen, Y. Shi, D. Chen, Y. Shi, Observation on the therapeutic effect of Shi's manipulation in treating cervical spondylotic radiculopathy, The Journal of Traditional Chinese Orthopedics and Traumatology (4) (2015) 12-14,20.

[13] W. Xu, Observation on the therapeutic effect of acupuncture and massage combined with rehabilitation in the treatment of cervical spondylotic radiculopathy, Global Traditional Chinese Medicine (s1) (2015) 21.

[14] S. Zhang, M. Wang, P. Wang, G. Mu, Clinical study on massage combined with electroacupuncture in the treatment of cervical spondylotic radiculopathy, Shaanxi Journal of Traditional Chinese Medicine (8) (2015) 1059-1060. DOI: 10.3969/j.issn.1000-7369.2015.08.058

[15] F. Jing, X. Niu, X. Wang, Y. Zhou, Clinical observation on penetrating moxibustion combined with electroacupuncture in the treatment of cervical spondylotic radiculopathy of wind-cold arthralgia type, Chinese Journal of Integrated Traditional and Western Medicine in Intensive and Critical Care 22(4) (2015) 367-369.

[16] Y. Zeng, Clinical efficacy of moxibustion combined with Canggui acupuncture in patients with cervical spondylotic radiculopathy, Shaanxi Journal of Traditional Chinese Medicine 37(4) (2016) 484-485. DOI: 10.3969/j.issn.1000-7369.2016.04.050

[17] H. Li, Y. Ma, Clinical observation on the treatment of cervical spondylotic radiculopathy with acupuncture combined with massage and traction, Journal of Sichuan Traditional Chinese Medicine 34(5) (2016) 193-195.

[18] H. Lin, Clinical observation on the treatment of cervical spondylotic radiculopathy by acupuncture combined with auricular acupuncture along the skin, Journal of Clinical Acupuncture and Moxibustion 32(5) (2016) 25-28.

[19] Y. Lin, Comparative observation of acupuncture and massage and posterior approach needle knife in the treatment of cervical spondylotic radiculopathy, Journal of Sichuan of Traditional Chinese Medicine 34(4) (2016) 187-189.

[20] L.a. Liu, S. Zhang, H. Wang, L. Tang, Observation on the therapeutic effect of acupuncture plus moxibustion in the treatment of cervical spondylotic radiculopathy, Chinese Acupuncture & Moxibustion 36(2) (2016) 139-143. DOI: 10.13703/j.0255-2930.2016.02.007

[21] C.e. Wang, Clinical study on electroacupuncture combined with massage in the treatment of cervical spondylotic radiculopathy, China Journal of Chinese Medicine 31(10) (2016) 1614-1618. DOI: 10.16368/j.issn.1674-8999.2016.10.454

[22] D. Wang, B. Rong, Clinical observation of a randomized controlled trial of abdominal massage combined with acupuncture in the treatment of cervical spondylotic radiculopathy, Liaoning Journal of Traditional Chinese Medicine 43(12) (2016) 2627-2630. DOI: 10.13192/j.issn.1000-1719.2016.12.055

[23] J. Wang, Z. Cui, J. Chen, Clinical observation on the treatment of cervical spondylosis with massage combined with lower cervical resistance exercise, Journal of Clinical Acupuncture and Moxibustion 32(11) (2016) 52-55. DOI: 10.3969/j.issn.1005-0779.2016.11.018

[24] D. Sun, Y. Chen, S. Zhou, H. Zhang, F. Sheng, Q. Lv, C. Ni, P. Cao, R. Cai, X. Qian, J. Zhang, H. Ma, A multicenter clinical study on electroacupuncture and massage combined with behavioral intervention in the treatment of cervical spondylosis, Shanghai Journal of Acupuncture and Moxibustion 36(9) (2017) 1092-1098. DOI: 10.13460/j.issn.1005-0957.2017.09.1092

[25] S. Zeng, Effects of acupuncture, massage and cervical traction on pain relief and hs-CRP, IL-8 and substance P in patients with cervical spondylotic radiculopathy, Journal of Hunan Normal University(Medical Sciences) 14(5) (2017) 46-49. DOI: 10.3969/j.issn.1673-016X.2017.05.014

[26] Y. Li, Z. Wei, Effects of traditional acupuncture and massage combined with McKenzie therapy on cervical function and electrophysiological indicators in patients with cervical spondylotic radiculopathy, The Journal of Cervicodynia And Lumbodynia 38(5) (2017) 420-423. DOI: 10.3969/j.issn.1005-7234.2017.05.004

[27] Z. Lin, J. Wang, Clinical observation on the treatment of senile cervical spondylotic radiculopathy by modified joint mobilization combined with massage, Geriatrics & Health Care 23(3) (2017) 223-225.

[28] S. Ping, Y. Zhang, H. Liu, C. Liang, Clinical study on the treatment of cervical spondylotic radiculopathy with Gutong plaster combined with nerve injury therapeutic apparatus, cervical vertebra massage and traction, Hebei Journal of Traditional Chinese Medicine 39(6) (2017) 858-862. DOI: 10.3969/j.issn.1002-2619.2017.06.014

[29] Q. Tang, Clinical observation on acupuncture combined with fingertip bloodletting for the treatment of cervical spondylotic radiculopathy, Journal of Sichuan Traditional Chinese Medicine 35(2) (2017) 178-179.

[30] M. Yao, Observation on the therapeutic effect of massage combined with electroacupuncture and simple traction in the treatment of cervical spondylotic radiculopathy, Journal of Traditional Chinese Medicine University of Hunan 37(7) (2017) 802-804. DOI: 10.3969/j.issn.1674-070X.2017.07.028

[31] Y. Zhang, Study on the therapeutic effect of acupuncture combined with massage in the treatment of cervical spondylotic radiculopathy, Shaanxi Journal of Traditional Chinese Medicine 38(8) (2017) 1126-1127. DOI: 10.3969/j.issn.1000-7369.2017.08.067

[32] Y. Cheng, T. Liu, B. Wang, L. Tang, Clinical Observation on the Treatment of Cervical Spondylotic Radiculopathy with Four-Needle Therapy plus Zheng's Manipulation, Chinese Journal of Traditional Medical Traumatology & Orthopedics 26(10) (2018) 40-44.

[33] Z. Ding, G. Zeng, Clinical study on deep needling of Dazhui acupoint combined with chiropractic massage in the treatment of cervical spondylotic radiculopathy, China Journal of Chinese Medicine 33(4) (2018) 676-681. DOI: 10.16368/j.issn.1674-8999.2018.04.161

[34] Y. Li, X. Wang, D. Wang, X. Chen, X. Yu, Y. Zhang, H. Wang, W. Yang, H. Wu, Clinical study on pain point aspiration in the treatment of cervical spondylotic radiculopathy, Journal of Emergency in Traditional Chinese Medicine 27(10) (2018) 1771-1773. DOI: 10.3969/j.issn.1004-745X.2018.10.022

[35] H. Tan, Comparison of the efficacy of trigger point acupuncture and conventional electroacupuncture in the treatment of cervical spondylotic radiculopathy, The Journal of Cervicodynia And Lumbodynia 39(5) (2018) 648-650. DOI: 10.3969/j.issn.1005-7234.2018.05.003

[36] Y. Tao, Y. Du, S. Tang, Q. Zhou, Observation on the therapeutic effect of acupuncture combined with cupping in the treatment of C5-6 single segment cervical spondylotic radiculopathy, Shanghai Journal of Acupuncture and Moxibustion 37(8) (2018) 932-936. DOI: 10.13460/j.issn.1005-0957.2018.08.0932

[37] H. Tian, C. Xiong, T. Dang, Analysis of the short-term and long-term efficacy of fire needle combined with bone setting massage in the treatment of cervical spondylotic radiculopathy, Journal of Clinical and Experimental Medicine 17(12) (2018) 1311-1315. DOI: 10.3969/j.issn.1671-4695.2018.12.024

[38] T. Wang, W. Wu, Q. Sun, Observation on the therapeutic effect of acupuncture at "three root points" combined with fine-tuning manipulation in the treatment of cervical spondylotic radiculopathy, Western Journal of Traditional Chinese Medicine 31(1) (2018) 112-114. DOI: 10.3969/j.issn.1004-6852.2018.01.033

[39] W. Wang, S. Qian, Y. Qi, Observation on the therapeutic effect of relaxation finger kneading method plus plucking the "Jiquan" acupoint combined with electroacupuncture in the treatment of cervical spondylotic radiculopathy, Guiding Journal of Traditional Chinese Medicine and Pharmacology 24(3) (2018) 113-114.

[40] Y. Zhu, M. Xu, Y. Huang, X. Ma, Z. Shi, S. Xu, H. Liu, S. Wang, H. Wu, Observation on the therapeutic effect of warm needling in treating cervical spondylotic radiculopathy, Shanghai Journal of Acupuncture and Moxibustion 37(12) (2018) 1413-1418. DOI: 10.13460/j.issn.1005-0957.2018.12.1413

[41] Y. Zhang, C. Limin, S. Pengfei, Z. Mengjiao, J. Hongjun, W. Lei, W. Yaochi, A randomized controlled study on the treatment of wind-cold-damp type of cervical spondylotic radiculopathy by Tongyang-Wenjing method, Journal of Hunan University of Chinese Medicine 38(0) (2018) 17-19.

[42] X. Zhang, J. An, W. Xie, Observation on the therapeutic effect of acupuncture combined with chiropractic therapy in the treatment of cervical spondylotic radiculopathy, Modern Journal of Integrated Traditional Chinese and Western Medicine 27(28) (2018) 3150-3153. DOI: 10.3969/j.issn.1008-8849.2018.28.022

[43] C. Gao, Z. Zhao, Evaluation of the effect of traditional Chinese medicine Fuyang cupping on cervical spondylotic radiculopathy, Chinese Nursing Research 33(21) (2019) 3767-3769. DOI: 10.12102/j.issn.1009-6493.2019.21.028

[44] J. Lv, Clinical observation on comprehensive treatment of cervical spondylotic radiculopathy with numbness as the main symptom, Hebei Medical Journal 41(6) (2019) 843-846. DOI: 10.3969/j.issn.1002-7386.2019.06.010

[45] S. Pan, S. Zheng, X. Zhou, Q. Wang, Acupuncture combined with Jingtong granules in the treatment of cervical spondylotic radiculopathy and its effects on IL-6, TNF-α, IL-1β and blood rheology indexes in patients, Chinese Acupuncture & Moxibustion 39(12) (2019) 1274-1278. DOI: 10.13703/j.0255-2930.2019.12.005

[46] Y. Tang, X. Li, J. Zhang, Effect of TCM syndrome differentiation and massage combined with acupuncture on cervical spondylotic radiculopathy, Journal of Sichuan Traditional Chinese Medicine 37(5) (2019) 187-190.

[47] K. Yang, Therapeutic effect of triple needling and warming acupuncture at cervical vertebral acupoints in the treatment of cervical spondylotic radiculopathy and its effect on cervical vertebrae mobility, Modern Journal of Integrated Traditional Chinese and Western Medicine 28(16) (2019) 1788-1791. DOI: 10.3969/j.issn.1008-8849.2019.16.021

[48] Z. Zhou, J. Zhu, W. Tang, B. Zheng, Clinical study on the treatment of neck arthralgia by needle knife whole body release combined with warm acupuncture and moxibustion, China Journal of Traditional Chinese Medicine and Pharmacy 34(8) (2019) 3854-3857.

[49] X. Guo, S. Kou, Observation on the therapeutic effect of deep acupuncture combined with chiropractic massage in the treatment of cervical spondylotic radiculopathy, Journal of Emergency in Traditional Chinese Medicine 29(1) (2020) 121-123. DOI: 10.3969/j.issn.1004-745X.2020.01.032

[50] X. Jiang, T. Zhang, X. Ren, Z. Wang, J. Lv, Y. Xu, W. Lin, X. Chang, Clinical study on the treatment of cervical spondylotic radiculopathy with three-dimensional bone setting and massage, Journal of Traditional Chinese Medicine University of Hunan 40(3) (2020) 337-341. DOI: 10.3969/j.issn.1674-070X.2020.03.018

[51] M. Li, X. Zhang, L. Xie, X. Ding, The efficacy of deep needling combined with massage in the treatment of cervical spondylotic radiculopathy and its effect on laboratory indexes, Anhui Medical and Pharmaceutical Journal 24(5) (2020) 881-884. DOI: 10.3969/j.issn.1009-6469.2020.05.008

[52] C. Song, Z. Lin, Clinical Observation on Electroacupuncture for Cervical Spondylotic Radiculopathy, Yi Shou Bao Dian 3 (2020) 0097.

[53] S. Zhang, T. Yao, Clinical analysis of ultrasound-guided needle knife release combined with acupuncture in the treatment of cervical spondylotic radiculopathy, Journal of Hubei College of Traditional Chinese Medicine 22(02) (2020) 102-105.

[54] Z. Guo, Q.e. Tan, G. Li, M. Li, H. Mo, Observation on the therapeutic effect of tendon-bone balance massage in treating cervical spondylotic radiculopathy, Journal of Emergency in Traditional Chinese Medicine 30(6) (2021) 1070-1073 DOI: 10.3969/j.issn.1004-745X.2021.06.035

[55] C. Jin, Treatment of cervical spondylotic radiculopathy with TCM chiropractic plus electroacupuncture combined with rehabilitation training, Journal of Changchun University of Traditional Chinese Medicine 37(5) (2021) 1044-1046. DOI: 10.13463/j.cnki.cczyy.2021.05.025

[56] JunhuMa, P. Hu, W. Zhou, Y. Zhou, Observation on the therapeutic effect of muscle bone balance massage in the treatment of cervical spondylotic radiculopathy, Western Journal of Traditional Chinese Medicine 34(4) (2021) 122-126. DOI: 10.12174/j.issn.2096-9600.2021.04.30

[57] LiangqunWu, ShuanglingLiu, TongjunLi, S. Shi, Effects of group acupuncture combined with chiropractic manipulation on cervical blood flow velocity, cervical function, pain mediators and plasma endothelin in patients with cervical spondylotic radiculopathy, Journal of Liaoning University of Traditional Chinese Medicine 23(10) (2021) 160-164. DOI: 10.13194/j.issn.1673-842x.2021.10.035

[58] X. Yu, Y. Wang, D. Zhang, E. Li, Observation on the therapeutic effect of deep needling of cervical Jiaji points with warm needles on cervical spondylotic radiculopathy, Journal of Chengdu Medical College 16(1) (2021) 82-86. DOI: 10.3969/j.issn.1674-2257.2021.01.020

[59] Q. Zhang, B. Shi, Cong'anWang, Z. Li, G. Sun, Clinical study on the treatment of cervical spondylotic radiculopathy with three-dimensional balanced spinal correction combined with acupuncture, Shandong Journal of Traditional Chinese Medicine 40(1) (2021) 65-70. DOI: 10.16295/j.cnki.0257-358x.2021.01.012

[60] YimoZhang, L. Zhang, C. Yu, L. Li, J. Zhou, Clinical Observation on the Treatment of Wind-cold-dampness Type Cervical Spondylotic Radiculopathy by Electroacupuncture at Jiaji Points Combined with Thunder-fire Moxibustion, World Journal of Integrated Traditional and Western Medicine 16(9) (2021) 1577-1580,1588. DOI: 10.13935/j.cnki.sjzx.210903

[61] X. Jin, R. Deng, W.H. Rong, Y. Wu, L. Xie, R. Kang, Clinical study on 40 cases of cervical spondylotic radiculopathy treated with traditional Chinese medicine bone setting manipulation combined with conventional therapy, Jiangsu Journal of Traditional Chinese Medicine 53(8) (2021 ) 44-48 DOI: 10.19844/j.cnki.1672-397X.2021.08.017

[62] L. Qiu, X. Leng, Warm acupuncture combined with nerve mobilization is effective in treating cervical spondylotic radiculopathy, Neural Injury and Functional Reconstruction 17(11) (2022) 678-681. DOI: 10.16780/j.cnki.sjssgncj.20210644

[63] J. Hou, B. Wang, P. Liu, J. Liu, J. Gao, J. Guan, X. Han, D. Sun, Effect of acupuncture combined with exercise therapy on cervical spondylotic radiculopathy and its effect on blood rheology, Journal of Clinical Acupuncture and Moxibustion 38(7) (2022) 20-23. DOI: 10.19917/j.cnki.1005-0779.022128

[64] J. Lan, Z. Liang, JiabaoSu, Y. He, HaojiaLin, Clinical study on the treatment of cervical spondylotic radiculopathy by massage manipulation combined with dragon-tiger acupuncture at cervical vertebral acupoints under the guidance of the idea of "giving equal importance to tendons and bones", Progress in Modern Biomedicine 22(15) (2022) 2888-2892. DOI: 10.13241/j.cnki.pmb.2022.15.017

[65] Y. Li, Y. Zhou, Observation on the therapeutic effect of dredging meridian points with massage combined with acupuncture in the treatment of cervical radiculopathy of qi stagnation and blood stasis type, Journal of Sichuan Traditional Chinese Medicine 40(11) (2022) 202-204.

[66] M. Ren, J. Shao, B. Li, J. Guo, H. Wu, Observation on the therapeutic effect of acupuncture combined with acupoint massage in the treatment of cervical spondylotic radiculopathy of qi stagnation and blood stasis type, The Journal of Cervicodynia And Lumbodynia 43(5) (2022) 745-747. DOI: 10.3969/j.issn.1005-7234.2022.05.039

[67] T. Yin, B. Luo, Q. Gao, Y. Wang, J. Zhang, Effect of suspension exercise therapy combined with massage and tendon-regulating manipulation on upper limb sensory function in patients with cervical spondylotic radiculopathy, Journal of Medical Biomechanics 37(1) (2022) 169-173. DOI: 10.16156/j.1004-7220.2022.01.026

[68] J. Chen, L. Li, N. Ning, Z. Yang, T. Li, Z. Yu, The efficacy of acupuncture combined with massage in the treatment of cervical spondylotic radiculopathy and its effect on patients' shoulder pain and cervical range of motion, Journal of Hubei University of Chinese Medicine 25(2) (2023) 90-92. DOI: 10.3969/j.issn.1008-987x.2023.02.24

[69] YuChen, XiaoleiDeng, Y. Wang, S. Wang, Evaluation of the therapeutic effect of acupuncture combined with manipulation in the treatment of cervical spondylotic radiculopathy, World Chinese Medicine 18(5) (2023) 677-681. DOI: 10.3969/j.issn.1673-7202.2023.05.016

[70] Q. Sun, X. Wang, Y. Yuan, W. Sheng, Observation on the efficacy of needle stimulation of painful points combined with ultrasound-guided nerve root block in the treatment of cervical spondylotic radiculopathy, International Journal of Anesthesiology and Resuscitation 44(2) (2023) 151-155. DOI: 10.3760/cma.j.cn321761-20220627-00731

[71] C. Zheng, Z. Luo, Y. Chen, Clinical efficacy of abdominal acupuncture combined with knot-untying acupuncture in the treatment of cervical spondylotic radiculopathy and its effect on neck and shoulder pain, Journal of Clinical and Experimental Medicine 22(5) (2023) 507-510. DOI: 10.3969/j.issn.1671-4695.2023.05.016

[72] L. Yi, X. Feng, J. He, Clinical study on acupuncture combined with meridian flow point massage in the treatment of cervical spondylotic radiculopathy, Henan Traditional Chinese Medicine 43(10) (2023) 1586-1590. DOI: 10.16367/j.issn.1003-5028.2023.10.0307

[73] A.-e. Hu, H.-q. Yang, Comparison of efficacy between electroacupuncture and the combination of collateral bloodletting, cupping, and acupoint application for cervical spondylosis radiculopathy (CRS), World Journal of Acupuncture - Moxibustion 24(3) (2014) 25-29. DOI: 10.1016/S1003-5257(15)60007-1

[74] J.-f. Zhou, X.-w. Li, J.-c. Zhao, L.-s. Wang, L. Wang, Y. Yang, Treatment of 30 patients with cervical spondylotic radiculopathy by acupuncture plus warming-needle moxibustion: a randomized controlled trial, World Journal of Acupuncture - Moxibustion 24(4) (2014) 24-28. DOI: 10.1016/S1003-5257(15)60023-X

[75] Y.-h. Bao, Randomized Controlled Study of Cervical Rehabilitation Training Combined with Acupuncture for Cervical Spondylosis Radiculopathy, Journal of Clinical Acupuncture and Moxibustion (2015).

[76] S. Wang, F. Sheng, Y. Pan, F. Xu, Z. Wang, L. Cheng, [Clinical study of cervical spondylotic radiculopathy treated with massage therapy combined with Magnetic sticking therapy at the auricular points and the cost comparison], Zhongguo Zhen Jiu 35(8) (2015) 773-7.

[77] Y. Ye, L. Xu, J. Yao, The Clinical Observation on Acupuncture Thermal Moxibustion Combined with Convention Acupuncture in Treatment of Acute Cervical Spondylotic Radiculopathy, Journal of Emergency in Traditional Chinese Medicine 24(6) (2015) 1091-1093. DOI: 10.3969/j.issn.1004-745X.2015.06.054

[78] L. Zhou, Q. Jiang, LihuaWu, D. Wu, Observations on the Efficacy of Combined Acupuncture and Medicine in Treating Cervical Spondylotic Radiculopathy with Syndrome of Wind-cold Obstructing Collaterals, Shanghai Journal of Acupuncture and Moxibustion (11) (2015) 1095-1098. DOI: 10.13460/j.issn.1005-0957.2015.11.1095

[79] L. Du, The Doctor of Traditional Chinese Medicine Massage Therapy Clinical Study on Cervical Spondylosis of Nerve Root Type, China Foreign Medical Treatment 35(31) (2016) 171-173. DOI: 10.16662/j.cnki.1674-0742.2016.31.171

[80] ZiyunFeng, L. Li, The Clinical Research into Cervical Spondylotic Radiculopathy Treated with Suspension Movement Therapy in Combination with Massage therapy, Henan Traditional Chinese Medicine 36(4) (2016) 696-698. DOI: 10.16367/j.issn.1003-5028.2016.04.0298

[81] F. Jing, X. Wang, X. Niu, Y. Zhou, Efficacy of Acupuncture Combined with Fire Dragon Moxibustion for Patients with Cervical Spondylotic Radiculopathy of Kidney-Deficiency-Cold Type, Acupuncture Research 41(4) (2016) 343-346. DOI: 10.13702/j.1000-0607.2016.04.011

[82] J. Li, Acupuncture-moxibustionand Massage in the Treatment of Cervical Spondylotic Radiculopathy 33 cases, Guangming Journal of Chinese Medicine 31(22) (2016) 3324-3325. DOI: 10.3969/j.issn.1003-8914.2016.22.044

[83] H. Zhang, Y. Wu, L. Shen, JunfengZhang, S. Zhang, T. Hu, Clinical study of neck point Bailao(Ex-HN15) Injection as main therapy plus acupuncture for cervical spondylotic radiculopathy, Shanghai Journal of Acupuncture and Moxibustion 35(10) (2016) 1238-1241. DOI: 10.13460/j.issn.1005-0957.2016.10.1238

[84] J. Wu, Y. Zhi, Y. Lv, Observation on clinical effects of acupuncture plus external medicine application for cervical radiculopathy, Journal of Acupuncture and Tuina Science 16(3) (2018) 171-175. DOI: 10.1007/s11726-018-1045-z

[85] X. Yang, L. Qian, L. Shen, J. Li, Clinical observation on warm needling moxibustion plus acupoint sticking therapy for cervical radiculopathy, Journal of Acupuncture and Tuina Science 17(2) (2019) 137-140. DOI: 10.1007/s11726-019-1103-1

[86] Y.-Z. Liu, L. Qu, X. Wang, W.-j. Xiong, W.-h. Li, H. Abula, J. Yang, S.-j. Zhai, Q.-h. Zhou, Effects of warm acupuncture combined with stereo-dynamic interferential electrotherapy on clinical efficacy and hemodynamics of cervical spondylotic radiculopathy, Int J Clin Exp Med 13(5) (2020) 3556-3563.

[87] P. Guo-liang, Z. Liang, D. Xiao-wei, Therapeutic efficacy observation of warm needling moxibustion plus spine subtle adjusting manipulation for cervical radiculopathy, Journal of Acupuncture and Tuina Science 19(6) (2021) 457-461. DOI: 10.1007/s11726-021-1283-3

[88] H. Shi, Q. Huang, W.P. Yao, L. He, C.D. Zhang, Z.M. Yang, [Randomized controlled trial on cervical spondylotic radiculopathy of wind-cold-damp type treated with acupuncture and thunder-fire moxibustion], Zhen Ci Yan Jiu 46(12) (2021) 1036-42. DOI: 10.13702/j.1000-0607.20210006

[89] X. Ji, L. Kong, Z. Gao, Y. Liu, H. Liu, Clinical observation of Jingfukang Granules combined with acupuncture in treatment of cervical spondylotic radiculopathy(wind-cold blocking collateral type), Chinese Traditional and Herbal Drugs 53(7) (2022) 2103-2107. DOI: 10.7501/j.issn.0253-2670.2022.07.020

[90] A.L. Li, X.W. Wang, J.R. Wang, F. Yu, Q. Li, H.N. Feng, L.S. Liu, W.G. Liu, [Clinical observation of acupotomy combined with warm needling for cervical spondylotic radiculopathy of qi and blood stagnation syndrome], Zhen Ci Yan Jiu 47(10) (2022) 914-6. DOI: 10.13702/j.1000-0607.20210968
